# Developing *Chenopodium ficifolium* as a potential B genome diploid model system for genetic characterization and improvement of allotetraploid quinoa (*Chenopodium quinoa*)

**DOI:** 10.1186/s12870-021-03270-5

**Published:** 2021-10-25

**Authors:** Madhav Subedi, Erin Neff, Thomas M. Davis

**Affiliations:** 1 Department of Biological Sciences, University of New Hampshire, Durham, USA; 2 Department of Agriculture, Nutrition, and Food Systems, University of New Hampshire, Durham, USA

**Keywords:** Quinoa, Allotetraploid, *Chenopodium ficifolium*, Diploid model system, Flowering time, *FTL*, Marker-trait association, Correlation

## Abstract

**Abstract:**

**Background:**

Quinoa (*Chenopodium quinoa*) is a high-value grain known for its excellent nutritional balance. It is an allotetraploid species (AABB, 2n = 4x = 36) formed by the hybridization between AA and BB genome diploid (2n = 2x = 18) species. This study reports genetic studies in *Chenopodium ficifolium* as a potential B genome diploid model system to simplify the genetic studies of quinoa including gene identification and marker-assisted breeding.

**Results:**

Portsmouth, New Hampshire and Quebec City, Quebec accessions of *C. ficifolium* were used to develop an F2 population segregating for agronomically relevant traits including flowering time, plant height, the number of branches, branch angle, and internode length. Marker-trait associations were identified for the *FLOWERING LOCUS T-LIKE 1* (*FTL1*) marker gene, where the alternate alleles (A1/A2) were segregating among the F2 generation plants in association with flowering time, plant height, and the number of branches. There was a strong correlation of the flowering time trait with both plant height and the number of branches. Thus, a possible multifaceted functional role for *FTL1* may be considered. The parental Portsmouth and Quebec City accessions were homozygous for the alternate *FTL1* alleles, which were found to be substantially diverged. SNPs were identified in the *FTL1* coding sequence that could have some functional significance in relation to the observed trait variation.

**Conclusion:**

These results draw further attention to the possible functional roles of the *FTL1* locus in *Chenopodium* and justify continued exploration of *C. ficifolium* as a potential diploid model system for the genetic study of quinoa. We expect our findings to aid in quinoa breeding as well as to any studies related to the *Chenopodium* genus.

**Supplementary Information:**

The online version contains supplementary material available at 10.1186/s12870-021-03270-5.

## Background

Quinoa (*Chenopodium quinoa* Willd.) is a “pseudo-cereal” grown for its edible seeds. It has an excellent balance of all of the essential nutritional elements and is considered a valuable source of nutrition for adults, infants, and children [1]. Quinoa is one of the best plant-based protein sources and is the only plant food that provides all ten essential amino acids required for the human body [1, 2]. Quinoa is also an excellent source of dietary fiber, and provides vitamins, minerals, and oils [3].

Quinoa breeding efforts are in progress worldwide, with the focus on higher yield, single stem, large grain size, disease resistance, early maturity, and low/no seedcoat saponin content [4]. Researchers at the New Hampshire Agricultural Experiment Station (NHAES) aim towards breeding or re-domesticating quinoa in Northern New England as a new crop option for regional farmers that will be highly nutritious and potentially highly profitable. However, local quinoa field trials thus far have produced disappointing results due to its poor adaptation to the local environment. For example, downy mildew disease, overly long maturation time, and immature grains at harvest are barriers for commercial cultivation of quinoa in New England. These and other agronomic constraints of quinoa such as excessive branching and severe lodging have also been reported by other researchers [5, 6].

Quinoa (*Chenopodium quinoa*), an allotetraploid with subgenome composition of AABB (2n = 4x = 36), is an evolutionary derivative of hybridization between AA and BB genome diploid *Chenopodium* species followed or accompanied by a chromosome doubling event [7–9]. This hybridization event is estimated to have occurred 3.3–6.3 million years ago in North America [10]. Besides quinoa, *C. berlandieri* Moq. and *C. hircinum* Schrad. also share the allotetraploid AABB subgenome composition, and these three species together form the AlloTetraploid Goosefoot Complex (ATGC) that went through a long-range dispersal, leading to the appearance and domestication of quinoa in the Lake Titicaca Basin of Peru and Bolivia [6, 9]. A phylogenetic study found *C. berlandieri* to be a basal member of the ATGC species complex, and it has been hypothesized that quinoa was descended from early tetraploids, specifically *C. berlandieri* via *C. hircinum* [10, 11]. The phylogenetic study of chloroplast and mitochondrial variants among *Chenopodium* species provides evidence for the A-genome diploid ancestor of quinoa to be the cytoplasmic donor in the original tetraploidization event [6].

The genome size of quinoa (1.45–1.5 Gb) and its transposable element content are postulated to comprise an additive combination of the ancestral diploid species genomes (0.6–0.9 Gb)  [12, 13]. The allotetraploidization has also resulted in gene duplication and fixation of heterozygosity in the genome of the resulting *Chenopodium* species [14]. These events have increased the genomic as well as computational complexity for study of the quinoa genome [13]. A marker-assisted breeding (MAB) program in quinoa would require various genomic resources such as a high-quality genome assembly, a genetic linkage map, and identification of marker-trait associations. But, due to its above-mentioned genomic complexities, genetic and genomic studies in quinoa are challenging [15, 16]. In contrast, genetic analysis in a diploid is comparatively straightforward, and thus the genomic mapping of a diploid relative can provide a relevant model system to identify associations between specific genes and agronomic traits of interest in a related polyploid like quinoa [17]. Thus, the AA and BB genome diploid *Chenopodium* species have potential as model systems to study the respective subgenomes of quinoa.

*Chenopodium ficifolium* (BB, 2n = 2x = 18), also known as fig-leaved goosefoot, is a weedy Eurasian species and is believed to be native to Southeastern Asia [18]. In a phylogenetic study of *SOS1* (*Salt Overly Sensitive* 1), *C. ficifolium* was the most closely related diploid species to the genome donor of the B homoeologs of quinoa [19]. Similarly, phylogenetic study of the *FLOWERING LOCUS T-LIKE* (*FTL*) genes among various *Chenopodium* species has indicated *C. ficifolium* to be a candidate for involvement in the hybridization events that produced ancestral quinoa [20]. Therefore, *C. ficifolium* is a highly appropriate candidate species to be explored as a potential diploid genomic model for gene-trait associations, and particularly those involving the B subgenome of allotetraploid (AABB) quinoa.

This study reports our foundational studies of *C. ficifolium* for the purpose of developing it as a model system relevant to quinoa genetic improvement. With focus on the quinoa breeding program and the agronomic constraints impeding its profitable cultivation in unfavorable environments, our main traits of interest were flowering time, plant height, internode length, number of branches, and branch angle. As discussed later, each of these traits is of agronomic interest in quinoa. Flowering time was considered to be a trait of particular relevance to quinoa cultivation in Northern New England, where early flowering and shortened time from seeding to harvest could be a solution to several production problems related to humidity and late seed maturation. Therefore, the possibility of identifying marker-trait, or even gene-trait association involving flowering time in *C. ficifolium* was of particular interest.

*FLOWERING LOCUS T-LIKE* (*FTL*) genes have been studied in quinoa, *C. berlandieri, C. ficifolium*, and other *Chenopodium* species [20, 21]. Three homologs of the *FT* gene have been identified: *FTL1*, which is orthologous to the sugar beet floral activator *BvFTL2*; as well as *FTL2-1* and *FTL2-2*, which are orthologous to the sugar beet floral inhibitor *BvFT1* [21]. However, the authors [21] also suggested the possibilities of the *FTL2* genes being involved in floral activation in the *Chenopodium* species, because their *FTL2* genes do not possess amino acid replacements that are associated to repressor function as in sugar beet [21]. Also, duplication of the *FTL2* gene happened more recently than the split between the *FTL1* and *FTL2* homologs [21]. Our study includes a major focus on the *FTL* gene in *C. ficifolium*, both because of its potential influence on an agronomic trait of interest and its usefulness as a molecular marker.

Recent genetic studies in the genus *Chenopodium* over the past few years have helped increase the availability of genetic resources. In 2012, a quinoa linkage map was developed that consisted of 29 linkage groups including putative SNPs identified among two recombinant inbred quinoa populations [22]*.* A phylogeny for *Chenopodium* species was developed based on the two introns of the single-copy nuclear locus *Salt Overly Sensitive 1* (*SOS1*) that helped to clarify the relationship among the genomes of the allopolyploid *Chenopodium* species and also to identify their subgenome donors [19]. As mentioned before, a phylogenetic tree was also developed among various *Chenopodium* species based on the introns of *FLOWERING LOCUS T-LIKE* (*FTL)*, where the authors found *FTL* to be a useful marker for tracking paternity in quinoa [20]. A genomic assembly of quinoa was developed in 2017 which provided invaluable information related to the subgenome sequences, and their gene content and location [10].

*C. pallidicaule,* a close A-genome diploid relative of quinoa, has also been sequenced and assembled [23]. Besides this, there is also a draft genome assembly of *C. suecicum,* a B-genome diploid relative of quinoa [10]. *Chenopodium* germplasm resources are maintained at 59 gene banks in 30 different countries, of which 80% are in the Andean region [4]. As of January 2020, the US National Plant Germplasm System has 370 accessions of *Chenopodium* that are publicly available, including 166 quinoa and four *C. ficifolium* accessions (www.ars-grin.gov/). Our aim is to contribute useful genetic, genomic, and germplasm resources in *C. ficifolium.* A preliminary report on this work has been presented in the Master’s thesis of M. Subedi [24].

## Methods

### Germplasm collection and maintenance

*C. ficifolium* germplasm was collected from two locations. The Portsmouth (P) accession (USDA accession: Ames 34,016) was collected from Portsmouth, NH (125 Bow St, 43^°^ 4.717′ N, 70^°^ 45.270′ W), and the Quebec City (QC) accession (USDA accession: Ames 34,020) was collected along the Saint Lawrence River in Quebec City, Quebec (46° 48.175′ N, 71° 12.449′ W). These two germplasm accessions were deemed to be acceptable for archiving in the USDA germplasm repository at Ames Iowa, and to our knowledge the collection was permissible, without requirement for a license, within the relevant framework of institutional, national, and international guidelines and legislation. The collected seeds were germinated in the Macfarlane Research Greenhouse facility at the University of New Hampshire (UNH), and individual plants were tested for species identification through morphological and molecular characterization. Seeds were collected from confirmed *C. ficifolium* plants of both the accessions and stored in paper envelopes at room temperature.

### Development of crossing methodology and hybridization between P and QC

Twenty seeds each of the P and QC accessions were sown in 72-cell trays with Pro-Mix substrate in a greenhouse mist room maintained at one mist cycle every 30 min. The seedlings were then transferred to a greenhouse maintained at 26 °C at 7 days after sowing (DAS). The seedlings were overhead watered twice per day manually and supplemented with Jacks 20-3-19 soluble fertilizer. At 15 DAS, the seedlings were transplanted to 10 cm diameter pots and were watered through drip water irrigation supplemented with the same fertilizer.

**Fig. 1:**
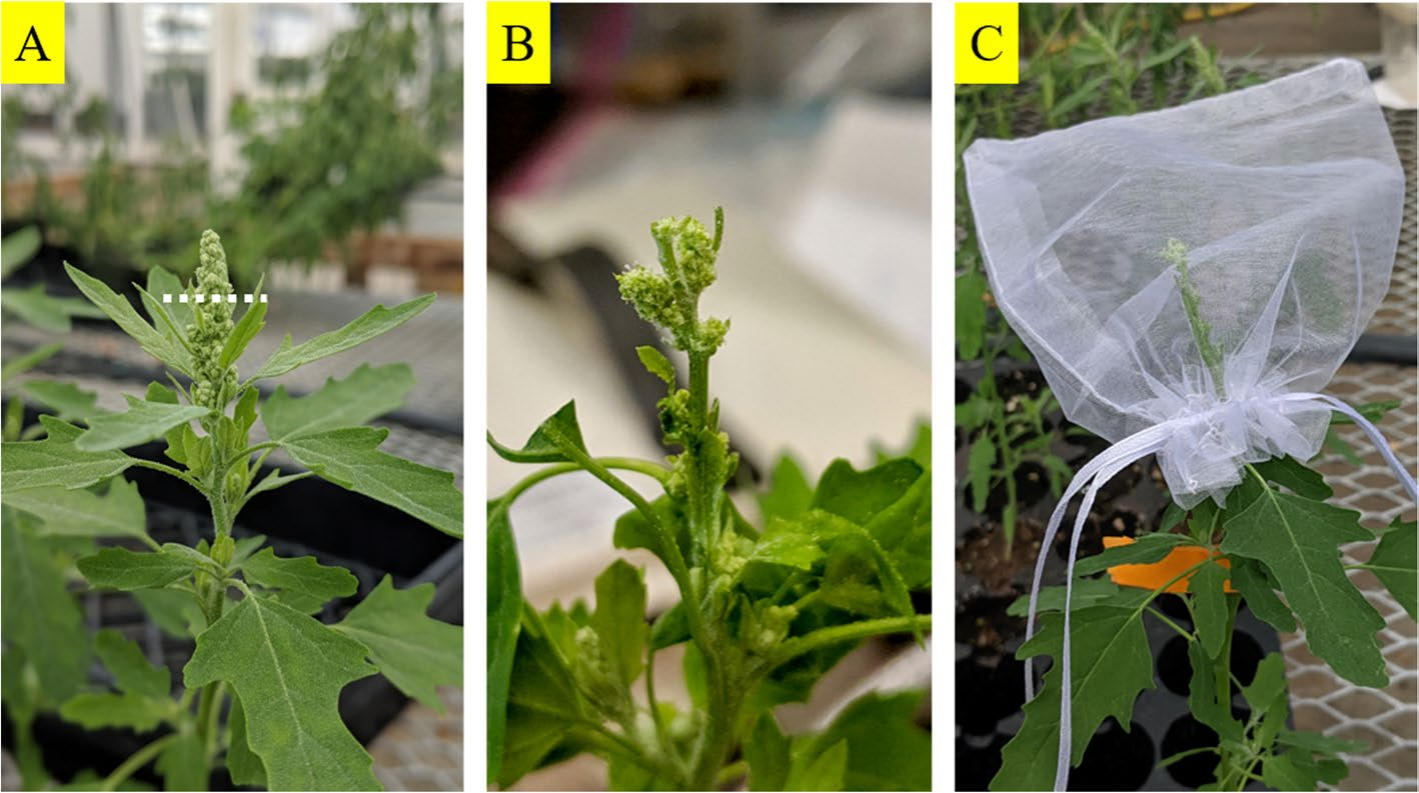
The process of cross-pollination in *C. ficifolium*. **A** shows an original inflorescence of a maternal parent with an indication of the main bud cut position and angle in white. **B** shows the same inflorescence after removal of its main flower bud, as well as the terminal flowers and surrounding leaves on the remaining lateral flower branches thus leaving behind a few pistillate flowers in the inflorescence for crossing. These pistillate flowers were cross-pollinated from the designated paternal parent, and then covered by an organza bag as seen in **C** to prevent unwanted cross-pollination

Two plants each of P and QC accessions were designated for use in crossing, and reciprocal crosses were made between the two accessions. To facilitate crossing, the main flower bud was cut off from the inflorescence of the maternal parent leaving the lateral flower clusters below the main flower bud (Fig. 1) to focus hybridization efforts on more manageable flower clusters as practiced in crossing quinoa [25]. The remaining flower clusters were viewed by magnification through a handheld magnifying lens and the terminal flowers which are mostly hermaphroditic were removed using sharply pointed forceps. Leaves surrounding the flower cluster were also removed. The final remaining pistillate flowers were then pollinated from the paternal parent through manual pollen dispersal by shaking of the paternal parent inflorescence over the maternal parent inflorescence. The inflorescence was then left covered with an organza bag (10.1 cm X 11.9 cm, candy/jewelry bags) as seen in Fig. 1 to prevent unwanted cross pollination from other plants as well as prevent any loss of seeds when matured. The inflorescence was observed for the course of the next 2 weeks after the crossing event for any missed hermaphrodite flowers, which if present were immediately removed. Subsequently, the putative hybrid seeds were collected after the seed maturation stage. The harvested seeds were then planted, and seedlings were grown in the manner previously described.

### Genotypic testing of putative F1 hybrids

Putative F1 hybrids were tested for hybridity in their vegetative stage using a PCR-based genotyping of the *FTL* marker [20, 26]. Five putative hybrid seedlings were tested from each of the crosses. DNA was extracted from each of the plants from green leaf tissue following a modified CTAB-based protocol [27] and was then quantified using an Invitrogen Qubit Fluorometer. The putative hybrids as well as their parental plants were genotyped by amplification of a segment of the *FLOWERING LOCUS T-LIKE* (*FTL*) gene using universal primers CrFT345For (5′-GGTTGGTGACTGATATTCCAG-3′) and CrFT501Rev (5′-CGCCACCCTGGTGCATACAC-3′) developed in a study of *Chenopodium rubrum* (*Oxybasis rubra*) [26]. Each 25 ul PCR reaction contained 0.4 μM of each primer, 2.5 units of LongAmp Taq Polymerase and 5 ul of 5X LongAmp Taq Reaction Buffer (New England Biolabs), 300 μM dNTPs (75 μM of each dNTP) and 25 ng of template. Gel electrophoresis-based fingerprinting of the PCR products was carried out on 1% agarose gels using a 1 Kb^+^ DNA ladder. Three different controls were used in the fingerprinting. A mixed template was prepared by adding template DNA from both of the parents in equal quantity to the pre-PCR reaction mix, while a mixed product control was prepared by adding together an equal amount of post-PCR product of each of the parents prior to gel loading Finally, a negative control was included where no template DNA was added to the pre-PCR mix. The thermocycler profile used for amplification was: initial denaturation phase of 94 °C for 30 s; 30 cycles of denaturation phase at 94 °C for 30 s, annealing at 54 °C for 1 min, and extension at 65 °C for 2 min; and a final extension at 65 °C for 10 min.

A putative hybrid was considered to be a true hybrid if it displayed *FTL* gel bands that were unique to each parent. The identified true hybrids from these crosses as well as from other crossing experiments were then selfed to produce F2 generation populations. To insure selfing, the identified true hybrids were kept in separate square net cages before the reproductive stage, at which point their inflorescences were covered with organza bags to enforce self-pollination and prevent any unwanted cross pollination.

### Characterization of *FTL* amplicons

For the purpose of obtaining a better understanding of the *FTL* marker system in *C. ficifolium*, amplicon bands were cloned or gel extracted and were sequenced**.** Cloning direct from PCR was performed using the TOPO TA Cloning kit for sequencing (Invitrogen) following its protocol. The isolated amplicons were then sequenced from both ends using the same *FTL* forward and reverse PCR primers, through the Eurofins (Eurofins Genomics LLC) sequencing service. Where needed to obtain a complete amplicon sequence, primer walking was done using forward and reverse sequencing primers that were designed using the initial forward and reverse sequences obtained from the initial round of amplicon sequencing. The DNASTAR (Lasergene, Ver.15) Primer Select program was used with default settings for designing the sequence walking primers, which were then obtained from Invitrogen. Sequence walking primers were designed such that each new sequenced read would contain overlap of at least 100 bp to the original sequence used for designing the primer and extend to amplify the remaining unsequenced region of the amplicon.

In order to confirm amplicon identity and differentiate between alleles of the previously differentiated [20, 21, 26] *FTL1* and *FTL2* loci, the amplicon contig sequences were then compared with each other to examine their sequence similarity using BLASTn. Each amplicon was also searched as a query against the entire *Chenopodium* genus database (taxid:3558), *C. ficifolium FTL1* intron3, (GenBank accession: KF910352) and *FTL2* intron3 (Gene bank accession: KF910328) to annotate the amplicons. The amplicons were also searched in the quinoa reference genome [10], a chromosome level assembly initially obtained from Dr. David Jarvis (personal communication), with identification number CQ41, which is now publicly available as the Cq_PI614886_V1 assembly (https://www.cbrc.kaust.edu.sa/chenopodiumdb/).

### Genotyping and segregation of *FTL1* marker genotypes in F2 population

For the purpose of confirming the presence of marker segregation in an expected Mendelian manner, the F2 generation plants were genotyped using the *FTL* marker system. The parental P and QC plants and putative hybrids were used as comparators to enable genotypic classification of the F2 segregants. Based on the segregating pattern of the identified *FTL1* marker alleles, the individuals of two F2 subpopulations were categorized into discrete genotypic classes, and the numbers of individuals in each of the genotypic classes were counted. A Chi-Square Goodness of fit test performed to see if the marker segregation patterns agreed or disagreed with the expected Mendelian segregation ratio of 1:2:1 for genotypes of a monohybrid cross. Furthermore, a homogeneity Chi-Square test was done to check if data from two subpopulations of the same F2 population could be pooled together for analysis. The X^2^ value of the pooled population, and the summed X^2^ of the two F2 subpopulations were used to calculate the homogeneity X^2^ value for testing the null hypothesis that the two F2 subpopulation were homogeneous [28].

### Trait diversity in parental accessions and their F2 generation

Only one of the several resulting F2 generation populations was evaluated during this study. Two experiments, designated as 1st and 2nd, were conducted consecutively in a Conviron PGR15 growth chamber set at 16 h. day, 8 h. night photoperiod with 22 °C day and 18 °C night temperature and relative humidity of 50%. The environmental regime was selected based on a preliminary study that was carried out to evaluate the parental accessions for differences in each of the traits as well as to identify an environment suitable for proper growth and easier visualization of phenotypic difference between the accessions (Table S1). During the 1st and 2nd experiment, a sufficient number of plants were directly seeded into individual 4″ diameter pots in the growth chamber. For the 1st experiment, seven plants each of the P and QC parental accessions, five F1 plants, and 26 F2 generation plants obtained from selfing of F1 hybrid P4F1D were randomly selected and were arranged following a Completely Randomized Design (CRD). For the 2nd experiment, four plants each of the P and QC parental accessions and 40 F2 plants were selected and arranged similar to the 1st experiment.

Each individual in the experiment was phenotyped with respect to flowering time, plant height, internode length, the number of branches, and branch angle. The same phenotyping techniques were followed in both the experiments. For flowering time, the plants were phenotyped at 2 day intervals until the first flower was observed, after which all plants were phenotyped every day. The plant height, number of branches, and branch angle were measured at 63 DAS for the 1st experiment and at 57 DAS for the 2nd experiment, by which time all plants within the experiment had reached the seed maturity stage. The plant height was measured from the surface of the potting mixture to the top of the central inflorescence. Notably, uniformity in the seed planting depth and the transplantation depth had been practiced to minimize inconsistencies in plant height measurement. For the number of branches, in order to exclude inflorescence branches from the count, the top 15% of the plant height (in cm) was excluded from the assessment, and branches were only counted from the remaining 85% of plant height. The mean internode length was calculated by dividing the plant height by the number of branches. Mean branch angle was calculated from the measured branch angles for the lowermost seven branches, per plant.

### Statistical analysis of trait diversity in parental and F2 generation

As detailed below, the distribution and t-test analysis were all done using JMP software (JMP Pro. 14.1 Statistical Software, Cary, NC) with default settings. Data for all of the traits were tabulated and stored in MS Excel. Calculations such as internode length and branch angle were done in MS Excel. A Shapiro-Wilk goodness-of-fit test for the normal distribution of each trait was done among the F2 generation population of both the experiments with a null hypothesis that the distribution was normal. For the rest of the analyses, a log transformation was done on the data for all of the traits to enable the data to follow a normal distribution. A t-test (two independent sample assuming unequal variance) was carried out between the P and QC plants grown in each of the experiments to test the null hypothesis that any difference between the mean trait values of P and QC accessions were attributable to chance alone. Mean values of the traits were reported after de-transformation.

### Correlation analysis of traits within two F2 subpopulations

Correlation between the phenotyped traits was tested using a multivariate correlation analysis where the traits were considered as separate variables. The test was done among the F2 generation population of both the 1st and 2nd experiment separately using the log transformed data. The obtained correlation r values were then used to identify any positive or negative correlation between the traits. The test was done using JMP software (JMP Pro. 14.1 Statistical Software, Cary, NC).

#### Association analysis of FTL1 genotypes with traits of interest

For the purpose of detecting possible associations between studied traits and the *FTL1* locus, log transformed phenotypic values of all the traits were assigned to F2 individuals belonging to each of the defined *FTL1* genotypes. Then a pairwise t-test was done for the comparison among the *FTL1* genotypic classes for each of the traits. The t-tests were done using JMP software (JMP Pro. 14.1 Statistical Software, Cary, NC) with default setting.

### *FTL1* genomic sequence divergence between P and QC accessions

As a first step toward illuminating a possible functional role for the *FTL1* locus, and ultimately other genes of interest, in influencing trait variation in *C. ficifolium*, the P and QC accessions were subjected to whole genome shotgun sequencing via Illumina HiSeq2500 into 250 bp paired end reads at the UNH Hubbard Center for Genome Studies (HCGS) (GenBank accession: PRJNA678370). Pseudochromosomes belonging to the B-subgenome of the quinoa Cq_PI614886_V1 assembly were concatenated into a separate assembly and referred to as B-subgenome-Cq_PI614886_V1. The P and QC reads were then aligned to the B-subgenome of the Cq_PI614886_V1 assembly using BWA mem. SNPs were discovered between the reference and each of the accessions using GATK (GATK 4.1.6.0) Haplotype caller and VCFtools (VCFtools 0.1.15) were used for indexing the VCF files. The two VCF files were then compared and SNPs unique to P and QC were identified by filtering the SNPs that were common between the accessions using BCFtools (BCFtools 1.7) isec and merge-SNPs parameters. The alignment was then used for identifying genetic divergence of the *FTL1* gene (AUR62006619) between each of the *C. ficifolium* accessions and quinoa.

De novo contig assemblies of P and QC Illumina reads were made using SPAdes (SPAdes 3.14.1). Then the genomic reads from each of the accessions were aligned to both contig assemblies using BWA mem (BWA 0.7.17). Each assembly was also searched for regions matching *C. ficifolium FTL1* mRNA, complete CDS (21, GenBank accession: MK212025), and *FTL* amplicon sequences of interest using BLASTn. The BLASTn and alignment was used for locating *FTL1* exon boundaries in the assemblies and to identify genetic divergence in the *FTL1* gene between the P and QC accessions. The CDS sequence of *C. ficifolium FTL1* (GenBank accession: MK212025), was aligned to the extracted *FTL1* gene of both P and QC contig assemblies as well as the *FTL1* gene of quinoa (GenBank accession: XP_021734330.1) to obtain a predicted protein alignment using Geneious prime (Geneious prime 2019.2.1) pairwise alignment with default settings.

## Results

### Crossing methodology in *C. ficifolium*

**Fig. 2:**
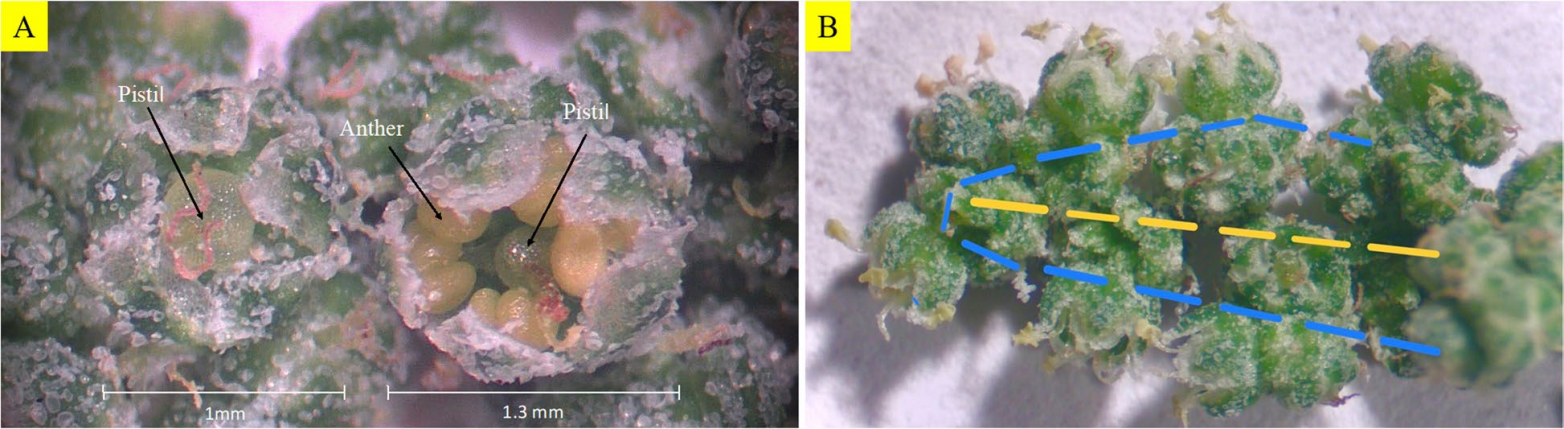
Flower and inflorescence morphology in *C. ficifolium***. A** Image of *C. ficifolium* inflorescences as observed through a dissecting scope, showing a female flower (left) and hermaphroditic flower (right). The size of the flowers is indicated by a scale bar. **B** Inflorescence of *C. ficifolium* observed under a dissecting scope. The yellow dotted line indicates the inflorescence branch placed horizontally with the top of the inflorescence facing left. The blue dotted line is drawn to delineate the locations of hermaphroditic flowers (ones with the yellowish anthers) on the distal region and periphery of the inflorescence

In its flower morphology, *C. ficifolium* was found to be similar to quinoa. The flowers are small and the inflorescence is of gynomonoecious nature (i.e. having both perfect and female flowers in the same individual, Fig. 2), which increases the difficulty of crossing using manual emasculation [29]. The hermaphrodite flower of *C. ficifolium* has 5 sepals and 5 antisepalous stamens, and a central ovary with multiple stigmas similar to quinoa (Fig. 2) [30]. The flower size of *C. ficifolium* is smaller than that of quinoa, which makes crossing even more time consuming and tedious. However, the inflorescence structure of both the *C. ficifolium* accessions was simpler than that of quinoa since the hermaphroditic/perfect flowers were mostly found as terminal flowers in the branching inflorescence as seen in the Fig. 2. The P plants tended to flower a week earlier than the QC plants.

### True hybrid identification

Ten to fifteen putative hybrid seeds were obtained from each of the crosses and five seeds/cross were planted to produce putative F1 hybrid plants for PCR testing. Amplification using the universal *FTL* primer pair [26] produced multiple bands in both P and QC. Some of these bands were monomorphic, being shared by both parents, and two bands were present in one parent and not the other (Figure S1). Specifically, the slower migrating band A1 was present only in parent P, while the faster migrating band A2 was present only in parent QC. Importantly, the “A” allele of the maternal parent was always present in the putative hybrids, but that of the paternal parent was present only in some putative hybrids. On this basis, presence of the paternally derived “A” allele in a putative hybrid was the criterion upon which hybridity was considered to be confirmed (Figure S1, arrows). Through this method, two true hybrids, P1F1A and P1F1C, were confirmed from the cross between P.1 (maternal parent) and QC4.1 (paternal parent) (Figure S1). Identification of true F1 hybrids was a prerequisite for advancing to development of a segregating F2 population by selfing.

### *FTL* genotyping and allelism testing in the F2 population

A total of 65 F2 plants were genotyped in two sequential experiments. The *FTL* genotyping of F2 generation plants generated multiple amplicons (Figure S2 and S3), similar to the F1 hybridization test results. Importantly, with respect to the marker bands A1 and A2, only three genotypes were represented: A1A1, A1A2, and A2A2. No F2 plant was lacking both A1 and A2 bands. Moreover, the Chi-Square Goodness of fit test results showed that genotypes A1A1, A1A2, and A2A2, were segregating in a Mendelian ratio of 1:2:1 in the F2 population of the 1st experiment (*P* > 0.95) as well as the 2nd experiment (0.7 > *p* > 0.5) (Table S2). The Chi-square homogeneity test on *FTL* segregation in the 1st and 2nd experiment F2 population samples indicated them to belong to the same population (0.80 > *p* > 0.70) (Table S2) and therefore they could be pooled together.

### Cloning and sequencing of *FTL* amplicons

With the aim of further defining the identities of *FTL* amplicons A1 and A2, cloning and gel extraction was then carried out to isolate the amplicons for sequencing. We were successful in isolating the five most prominent *FTL* generated amplicons for both the parental accessions (Figure S4). These included the segregating amplicons A1 and A2, which were around 2600 bp and 1500 bp, respectively, as determined by comparison with the DNA ladder. The other three sequenced amplicons B, C, and D were approximately 1350 bp, 1650 bp, and 850 bp in length, respectively. Amplicon A1 had to be gel isolated for sequencing since we were unable to clone it (Figure S4). Sequencing quality scores of higher than 45 were obtained for almost all of the amplicons except the reverse end read of amplicon C which had an average quality of around 25. However, further sequencing of amplicon C was not required as its forward sequence covered most of the amplicon length. Primer walking was necessary to obtain the full sequence of amplicon A1 because its forward and reverse end reads, which differed in length by around 500 bp, had no overlap. Sequencing primers were designed using initial forward and reverse sequences. For each amplicon, all of its sequence reads were assembled to generate a single contig representing the respective amplicon. The length of each contig was found to be similar to the respective amplicon’s size as estimated in comparison to the gel ladder.

### Annotation of the *FTL* amplicons and delineation of an *FTL* marker locus

In the following, GenBank sequences beginning with MW are from the present study while those beginning with KF [20] and MK [21] are from other studies of *Chenopodium* species. The forward and reverse sequences of each *FTL* amplicon were assembled to generate a contig for each amplicon using Geneious prime pairwise alignment (Geneious prime 2019.2.1) (our GenBank accession: MW251945 MW251946, MW251947, MW251948, MW251949 for A1, A2, B, C and D contigs, respectively).

#### Sequencing similarity among the FTL amplicons

**Fig. 3:**
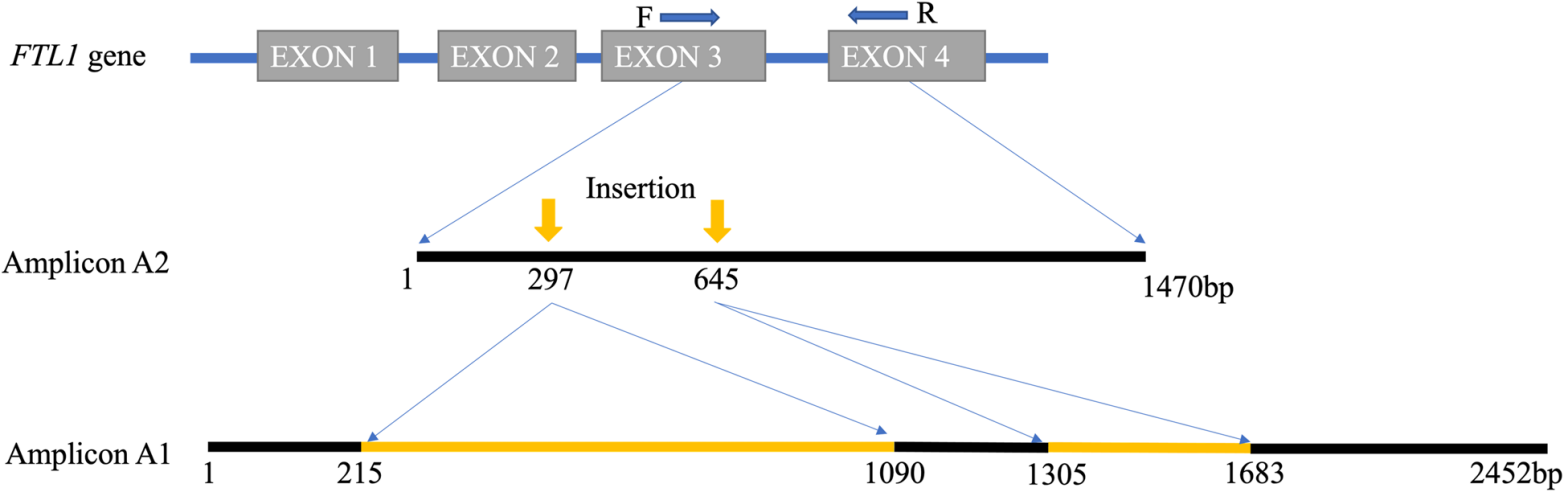
Insertional differences between *FTL1* amplicons A1 and A2. Illustration of differences between the A1 and A2 amplicons. In the figure, F and R bracket the amplification region of the *FTL1* gene from forward to reverse *FTL* primers, black segments indicate sequence similarity between the amplicons, yellow segments in the A1 amplicon indicate insertion and the yellow arrows above the A2 amplicon indicate the insertion position in the A2 amplicon relative to the A1 amplicon. The black segments also include small indels that contribute to minor differences in length of indicated segments between the two amplicons. The short, unique sequences found uniquely in the A2 amplicon are not illustrated in this figure

The BLASTn comparisons between the amplicons showed that the amplicons B, C and D (our GenBank accessions MW251947, MW251948, and MW251949, respectively) were unrelated to each other and to the amplicons A1 and A2 (our GenBank Accession: MW251945 and MW251946, respectively). The amplicons A1 and A2 both include the sequence region from exon 3 to exon 4 of the *FTL1* gene within the Cq_PI614886_V1 quinoa genome assembly. The amplicons A1 and A2 had approximately 1300 bp in common with an 88% identity between the two sequences. The difference between A1 and A2 amplicons was mainly due to two blocks of unique sequence found in amplicon A1 totaling approximately 1250 bp, suggestive of two insertion events in intron 3 of the P accession as illustrated in Fig. 3. These inserted sequences were searched in the *C. quinoa* B-subgenome *FTL1*-intron3 and partial CDS (GenBank accession: KF910363.1) and *C. ficifolium FTL1*-intron3 and partial CDS (GenBank accession: KF910352.1) [20], but we found no relation between the A1 inserts and the *FTL1* sequence of either quinoa or *C. ficifolium* GenBank accession KF910352.1 The A2 amplicon also had a total of 227 bp of unique sequences within its intron 3 region that were absent in the A1 amplicon sequence but present in *FTL1*-intron 3 of quinoa (GenBank accession: KF910363.1) as well as in a previously studied accession of *C. ficifolium* ([20]; GenBank accession: KF910352.1).

#### Sequence characterization of FTL amplicons

Both the amplicons A1 and A2 through BLASTn comparison showed sequence similarity to *FTL1*-intron 3 and partial CDS of many *Chenopodium* species including *C. quinoa* (GenBank accession: KF910363.1), *C. berlandieri* (GenBank accession: KF910362.1), and a *C. ficifolium* accession (GenBank accession: KF910352.1) [20]. About 45% of the A1 amplicon sequence and 75% of A2 amplicon was shared with the publicly available *FTL1* gene sequences of *Chenopodium* species. BLASTn search of the A1 amplicon to the Cq_PI614886_V1 quinoa reference genomes showed that about 70% of the amplicon was present in Chromosome 05 with greater than 80% identity (Table S3). Similarly, more than 95% of the A2 amplicon was found at the same location within Chromosome 05 (Table S3). Chromosome 05 has been annotated as belonging to the B-subgenome of quinoa, and it includes the *FTL1* gene (AUR62006620) [10]. Both amplicons had one or more blast hits in Chromosome 12 of quinoa (A-subgenome) ranging up to about 400 bp with about 80% sequence identity (Table S3). Therefore, these results indicated the A1 and A2 amplicons to belong to the *FTL1* gene of P and QC accessions of *C. ficifolium.*

Amplicon B had greater than 90% of its sequence covered by the *FTL2* gene (intron 3 and partial CDS) of *C. ficifolium* (GenBank accession: KF910328.1) and *C. quinoa* (GenBank accession: KF910340.1). BLASTn search of the amplicon to Cq_PI614886_V1 quinoa reference genome showed that the amplicon with greater than 90% coverage and percentage identity of greater than 95% was present in Chromosome 17 (Table S3) which is annotated to contain the *FTL2* gene and belongs to the B-subgenome in the quinoa genome assembly (Table S3). Amplicon sequence was also found in Chromosome 15, which is a part of the quinoa A subgenome. These findings indicate that amplicon B belongs to the *FTL2* gene of P and QC accessions of *C. ficifolium.* The amplicons C and D had no relation to any of the *Chenopodium FTL* gene sequences. The BLASTn search of the amplicons to the quinoa genome showed that amplicon C with 90% of query coverage and parentage identity of 94% was present in Chromosome 06, whereas, amplicon D with 95% of the coverage and a percentage identity of 98% was found within Chromosome 03 (Table S3). Both Chromosomes 03 and 06 belong to the B-subgenome of quinoa and amplicons sequence was also found to be present within A-subgenome chromosomes.

### Trait diversity in parental and F2 generation population

**Fig. 4:**
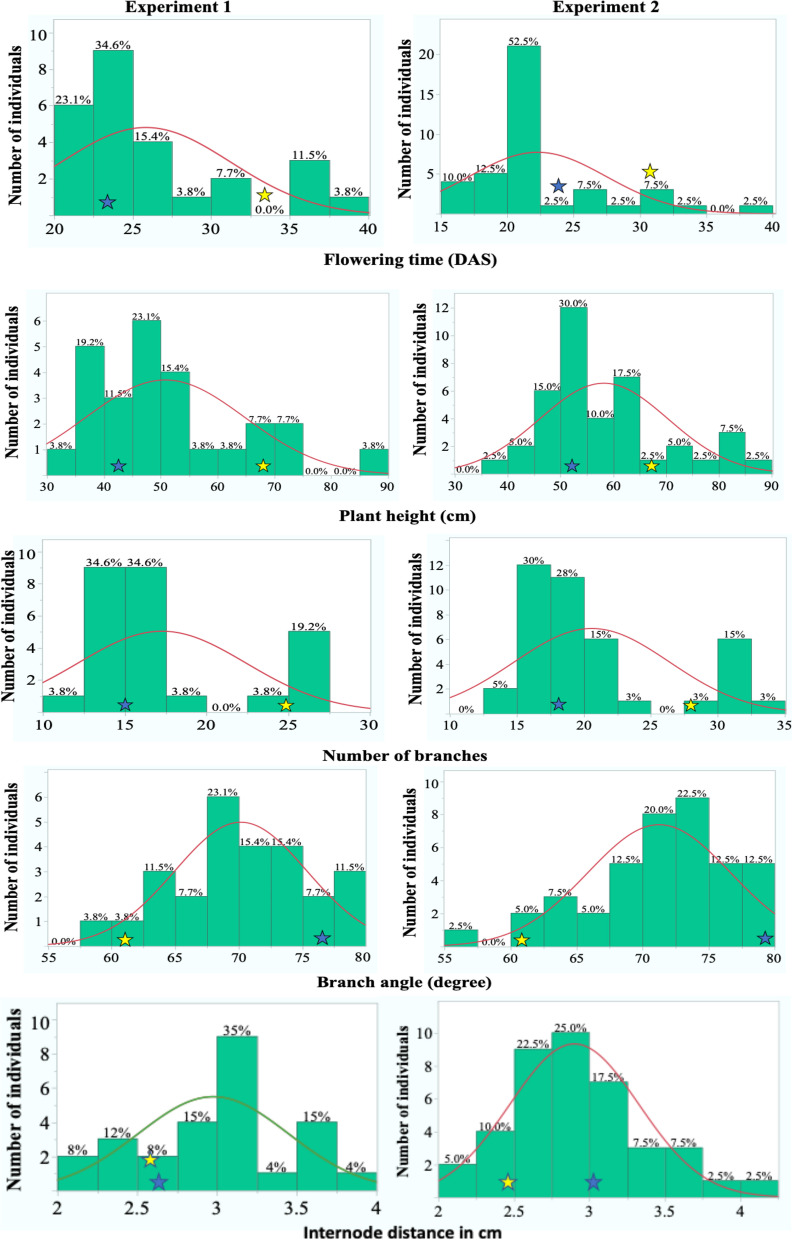
Traits segregation in F2 population of *C. ficifolium*. Distribution of flowering time, plant height, number of branches, branch angle and internode length among the F2 generation population in the 1st experiment (left) and the 2nd experiment (right). The X-axis represents the trait values and the Y-axis represents the number of individuals. The value at the top of each bar represents the percentage of individuals within that range of trait value. The red line indicates the trait distribution curve for the population. The mean value of traits for P and QC parental control plants is represented by a blue and a yellow star, respectively, along the X-axis for each of the experiments

Phenotypic trait diversity was first evaluated between the parental accessions since the chances of segregation of a trait in an F2 generation population would be higher if the parents are phenotypically diverse for the trait. Details on the phenotypic trait values of P and QC can be found in Table S4 as well as in distribution graphs of Fig. 4. The P and QC plants had significant differences in respect to flowering time, plant height, number of branches, and branch angle in both of the experiments (*p* < 0.03, Table S4). The P plants were earlier flowering, and had shorter stature, fewer branches, and wider branch angle than the QC plants in both experiments. However, the internode length had inconsistent results in the two experiments, where the P and QC plants were found to be significantly different in the 2nd experiment (*p* = 0.016) but not in the 1st (*p* = 0.32, Table S4). The same patterns of phenotypic variations were found for all of the traits in the preliminary study of the parental accessions (Table S1).

**Fig. 5:**
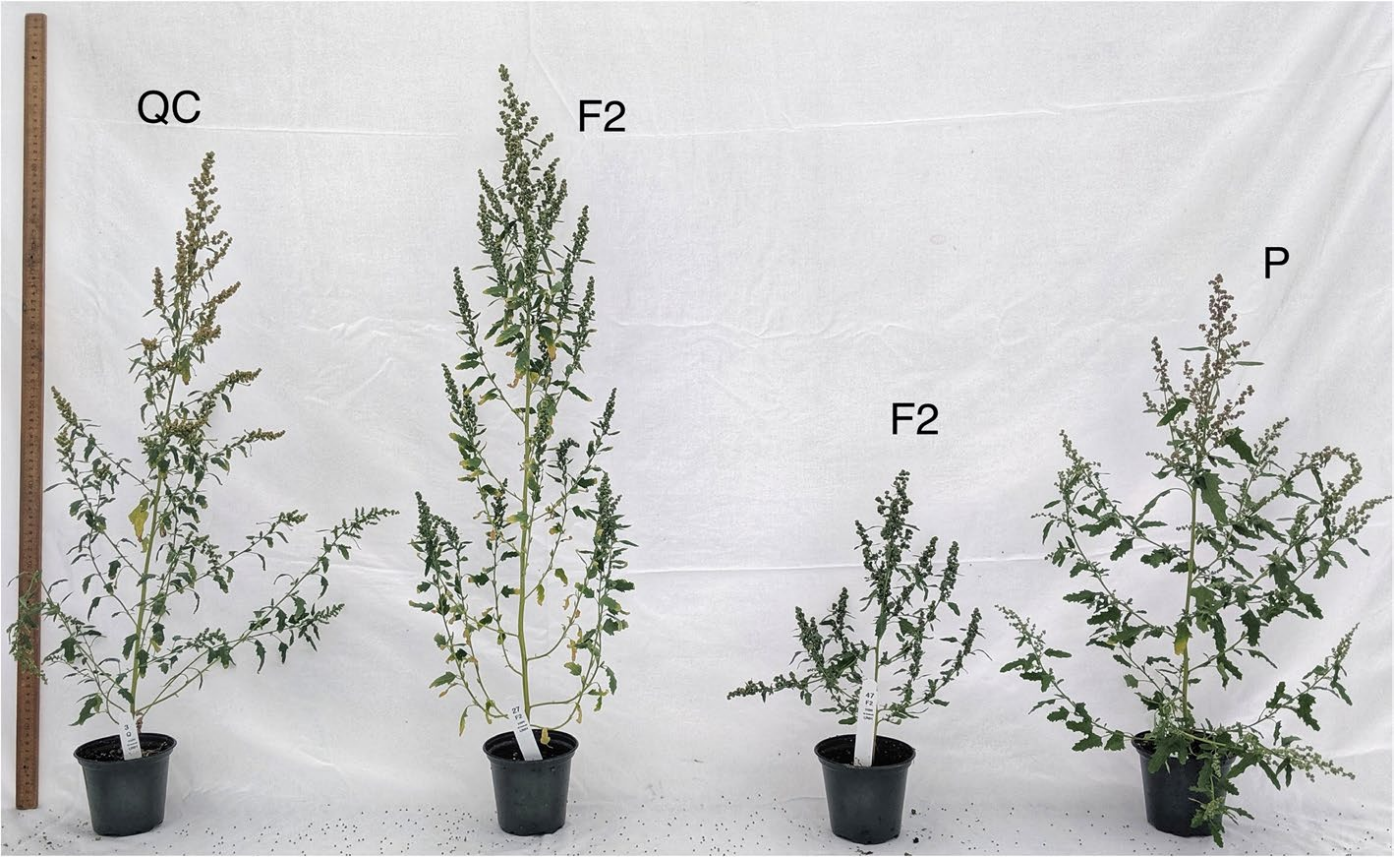
Transgressive segregation for plant height in F2 population. Illustration of plant height variation among the P, QC and two F2 generation plants grown in the 2nd experiment showing extreme phenotypes

The F2 generation population was then evaluated phenotypically for segregation of the traits. During the 1st and 2nd experiment, the F2 generation plants were found flowering as early as 16 DAS to as late as 39 DAS, with approximately 55–60% of the F2 population flowering in a time range of 20–25 DAS (Fig. 4). A non-normal distribution of flowering times was found in the F2 population of the 1st (*p* = 0.0001) as well as the 2nd (*p* = 0.0009) experiment. The F2 populations of both the 1st and 2nd experiment had plant heights ranging from 35 cm to 90 cm (Fig. 4), and the distribution was non-normal in both the experiments (*p* = 0.02). More than 65% of the F2 plants had plant heights less than 60 cm in both of the experiments. The difference in plant height between the parental controls and the extreme plant height expression (transgressive segregation) in the F2 plants is illustrated by the plants in Fig. 5.

Among the F2 plants of the two experiments, the number of branches varied from as low as 10 to as high as 35 (Fig. 4). More than 60% of F2 plants had branch numbers less than 20, and there were only about 20% of plants having more than 25 branches in both the experiments. The number of branches was non-normally distributed in the F2 population of both the 1st and 2nd experiment (*p* < 0.0001). The F2 plants in the 1st and 2nd experiment had branch angles ranging from approximately 55° to 80° (Fig. 4). The F2 population of the 1st experiment had a normal distribution pattern (*p* = 0.87) of branch angle, whereas the 2nd experiment had a non-normal distribution (*p* = 0.03). The F2 plants in both of the 1st and 2nd experiments had internode lengths varying from 2 cm to 4 cm (Fig. 4). The internode length was normally distributed in both experiments; *p* = 0.80 in 1st experiment and *p* = 0.44 in 2nd experiment.

### Correlation analysis of traits

**Fig. 6:**
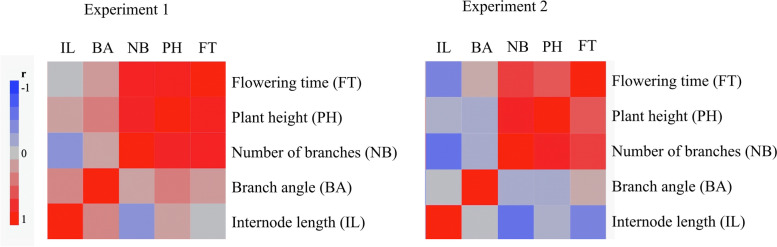
Correlation between traits in the F2 population. Color maps showing multivariate correlation test results between the traits phenotyped in the 1st (left) and 2nd experiment (right). The legend on the left of figure indicates the correlation coefficient (r) values for each of the representing colors. The color map was created using JMP software (JMP Pro. 14.1 Statistical Software, Cary, NC)

The multivariate correlation analysis between all the phenotyped traits of the 1st and 2nd experiments is presented in Fig. 6 and Table S5. The flowering time had a high positive correlation with the number of branches in both experiments (*r* > 0.74). A similar correlation was found between flowering time and plant height in the 1st experiment (*r* = 0.85) (Fig. 6 and Table S5). However, only a moderately positive correlation was found between flowering time and plant height in the 2nd experiment (*r* = 0.6). Plant height and the number of branches had a high positive correlation in both experiments (*r* > 0.8). The number of branches and internode length had a low to moderately negative correlation in the 1st (*r* = − 0.35) and 2nd (*r* = − 0.64) experiment. The remaining correlations between the traits were found to be low (Fig. 6 and Table S5).

### Association analysis of *FTL1* genotypes to traits of interest

**Fig. 7:**
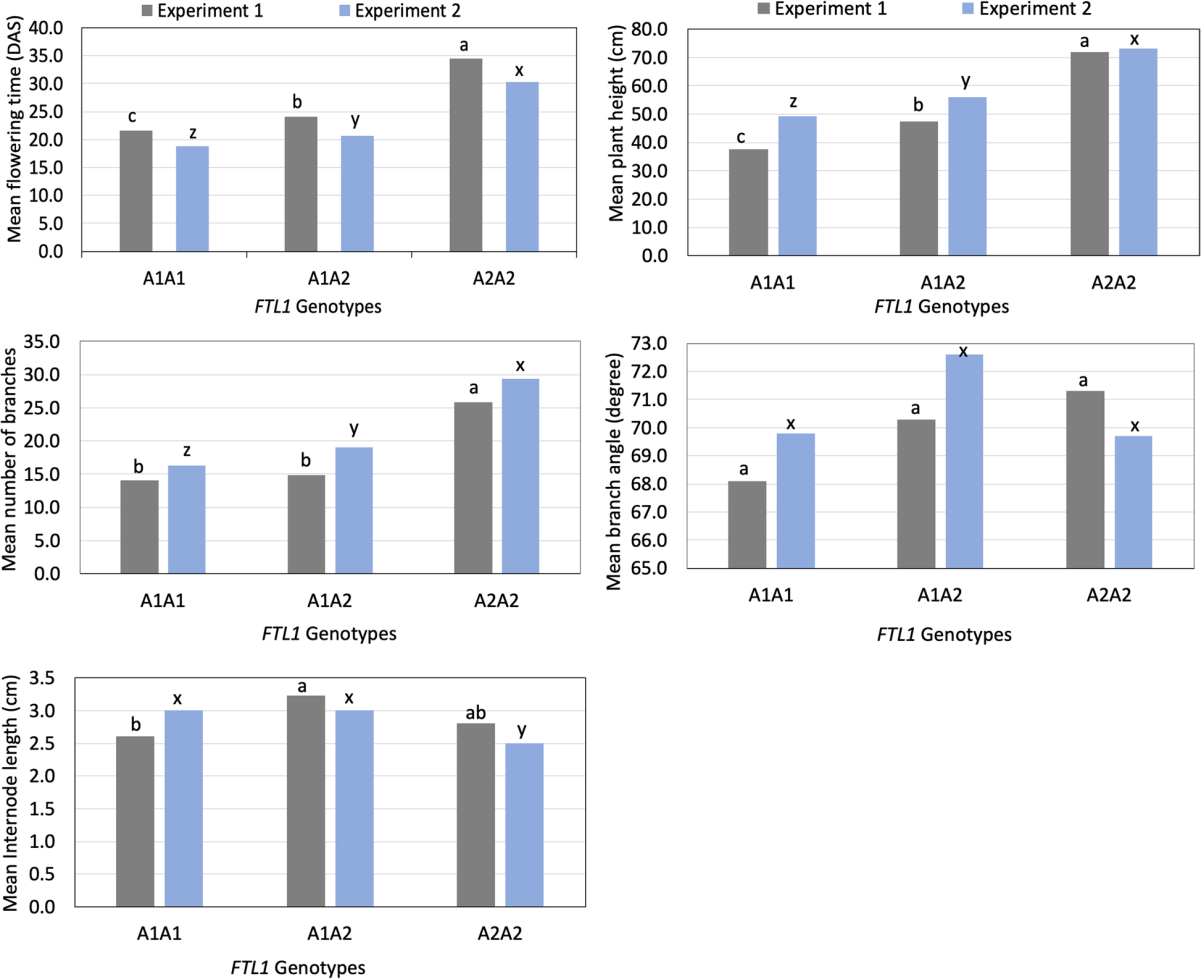
Marker-trait association in F2 population. Mean values of traits for three different *FTL1* genotypes A1A1, A1A2, and A2A2 in the 1st experiment (Gray) and 2nd experiment (blue). Pairwise t-test results between the genotypes are represented by letters a, b, and c for the 1st experiment and x, y, and z for the 2nd experiment where genotypes with different letters within an experiment represent a significant difference (*p* < =0.05)

As the F2 population were segregating for the *FTL1* alleles, we then wanted to see whether the *FTL1* genotypes had any association to our traits of interest. Our results showed that the *FTL1* genotypes A1A1, A1A2, and A2A2 had mean flowering times of 22, 24, and 35 DAS, respectively, in the 1st experiment, and of 19, 21, and 31 DAS, respectively, in the 2nd experiment (Fig. 7, Table S6). The A1A1 genotype had the lowest, A1A2 had the intermediate, and A2A2 the highest mean values for flowering time, plant height, and the number of branches in both the experiments (Fig. 7). The pairwise t-test comparison showed that each of the *FTL1* genotypes differed significantly from the other two (*p* < 0.02) for flowering time, plant height, and the number of branches, with one exception (Fig. 7, Table S6). We found no significant difference between the A1A1 and A1A2 genotypes for the number of branches in the 1st experiment (*p* = 0.37). The branch angle was not significantly different among the genotypes in either experiment (*p* > 0.1). For internode length, the A1A1 genotype was found significantly different from the A1A2 genotype (*P* = 0.03) in the 1st experiment, whereas the A2A2 genotype was found significantly different from the other two genotypes in the 2nd experiment (*p* < 0.002). The rest of the genotypic comparisons had no significant differences in internode lengths (Fig. 7, Table S6).

### *FTL1* genomic sequence divergence between P and QC accessions

With P and QC being found to have phenotypic diversity for these several agronomic traits, our interest was also to begin to explore genomic divergence between these accessions, with particular emphasis on the *FTL1* locus. Based upon the analysis of Illumina read data that we generated from the P and QC accessions (GenBank accession: PRJNA678370), a total of 5,218,465 unique SNPs were identified between the P and QC accessions in reference to the B-subgenome-Cq_PI614886_V1 pseudochromosome assembly. Also with the accessions being found to contain alternate alleles of the *FTL1* gene, our interest lay in identifying genetic divergence for this gene between these accession. For this, de novo contig assemblies of each accession were constructed using the Illumina read data, and the assemblies were then used to locate and identify the amplicon regions and the entire *FTL1* gene sequences in each accession. For the P accession, a contig level assembly of length 711 Mb was generated, with a contig N50 of 20,061 bp, average read depth of 26x, and GC% of 36.56. Similarly, the QC accession contig assembly had a length of 770 Mb with a contig N50 of 18,535 bp, average read depth of 26x, and GC% 36.59. BLASTn showed that the entire *C. ficifolium FTL1* transcribed region and complete CDS (GenBank accession: MK212025) including the A1 amplicon sequence was present in a single contig of the P assembly. This contig also included the downstream neighbor gene (AUR62006620) of *FTL1* from the Cq_PI614886_V1 quinoa assembly [10]*.* However, for the QC assembly, the *FTL1* transcribed region was divided between two separate contigs, while we found the entire A2 amplicon within a single contig.

**Fig. 8:**
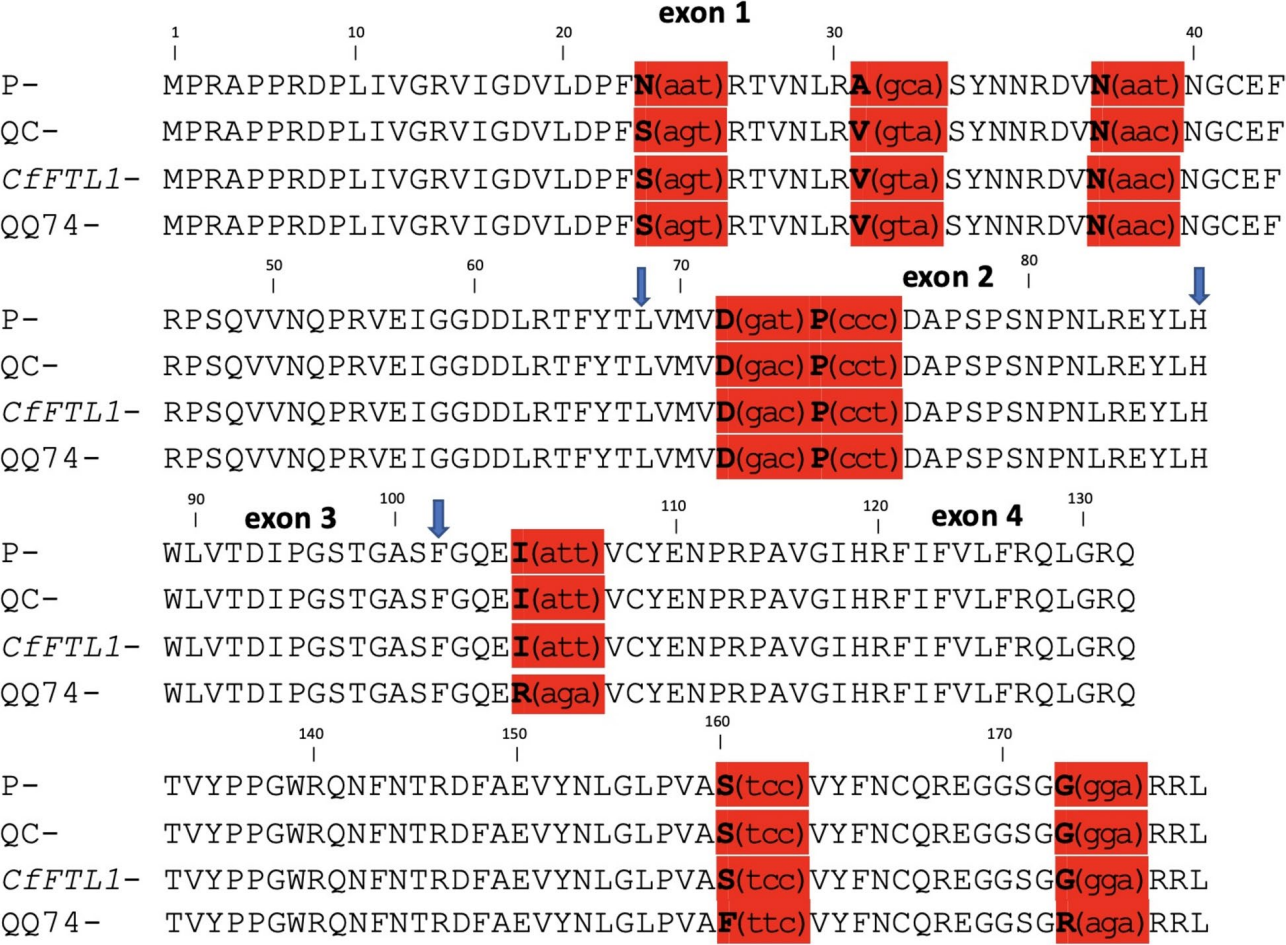
Sequence polymorphisms in *FTL1* predicted protein sequence. Alignment of *FTL1* protein sequence of P, QC, *CfFTL1* ([21], GenBank accession:MK212025), and Quinoa (GenBank accession: XP_021734330.1). The exon boundaries of *FTL1* are indicated by blue arrows. The synonymous and non-synonymous substitution are highlighted by bold letters of amino acid within the red blocks. The codon sequences for these amino acids are given within parenthesis on their right

**Fig. 9:**
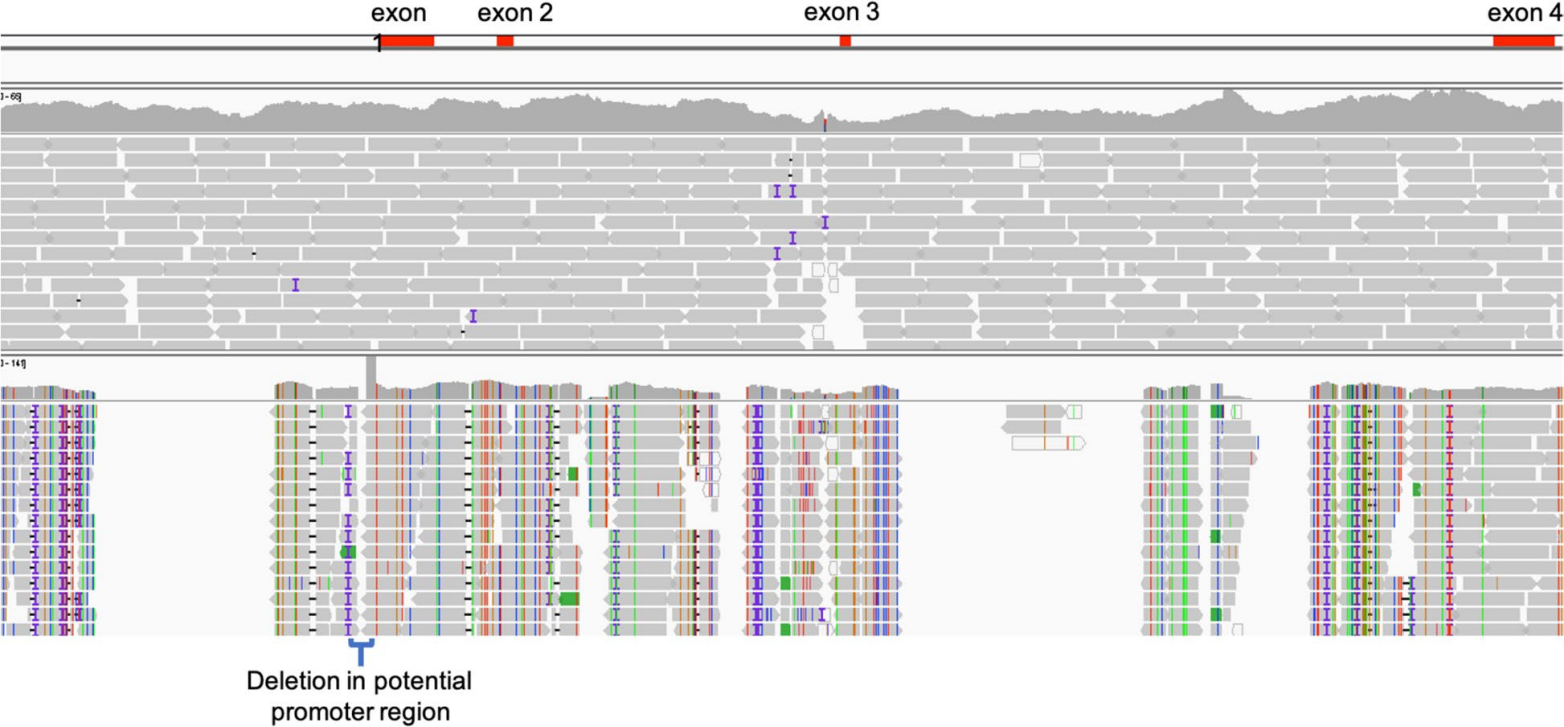
DNA sequence polymorphisms in *FTL1* gene. Illumina reads from P (top) and QC (bottom) aligned to the P contig containing the full *FTL1* gene, as displayed using Integrative Genomic Viewer (IGV Linux 2.9.4 With Java), showing polymorphism between P and QC in the *FTL1* gene. The *FTL1* exons are represented by four red boxes on the top track. The bracket indicates a 30 bp deletion, including a TATA sequence within the likely promoter region

We found 107 SNPs between the accessions P and QC in the *FTL1* gene transcribed region during initial variant calling in relation to the B-subgenome-Cq_PI614886_V1 pseudochromosome assembly (Table S7). We did not find any evidence of heterozygosity in either accession for the amplicon sequence region or the entire *FTL1* gene. During the comparison of P and QC through alignment to the P contig assembly, we identified five SNPs in CDS sequences of the *FTL1* gene (Fig. 8, Table S7). Three of these SNPs were in exon 1 (CDS) and two in exon 2, of which two resulted in non-synonymous substitution and the remaining three were synonymous substitutions (Fig. 8). The *FTL1* gene of a previously studied *C. ficifolium* accession [21] (*CfFTL1* mRNA, complete CDS, GenBank accession: MK212025) was found to be a perfect match to our QC accession *FTL1* gene sequence. We also found that quinoa *FTL1* protein sequence ([10], GenBank accession: XP_021734330.1) was similar to the QC accession for the above-mentioned substitutions but it also includes unique SNPs in exon 4 that result in three non-synonymous substitutions (Fig. 8). The P and QC accessions also have a substantial difference in promoter and intron sequence of the *FTL1* gene as seen in Fig. 9. The QC accession had a deletion located in close proximity to the upstream end of the *FTL1* transcribed region, and this deletion encompassed a possible TATA box region. This site could be a potential promoter region present in the P accession but missing in QC. We also found a second potential promoter site upstream of the deletion region that was present in both accessions. These results indicated that *FTL1* genes of P and QC accessions of *C. ficifolium* are genetically divergent in ways that might have functional significance.

## Discussion

### Towards a B genome diploid model system for quinoa

The genetic study of a tetraploid species is much more complex than the study of its diploid ancestors [17]. The present study focused on *C. ficifolium*, a diploid of genome composition BB, to initiate its development as a potential model system for the genetic characterization and improvement of allotetraploid (AABB) quinoa. An important goal of our genetic research on *C. ficifolium* is to develop a B genome linkage map containing markers associated with polymorphic traits in *C. ficifolium* that are of agronomic interest in quinoa. This investigation constitutes a substantial step toward the accomplishment of this goal by defining suitable parents, generating F1 and F2 populations, and documenting trait segregation and marker-trait association in an F2 population.

### Traits of interest

As an ideotype for quinoa breeding in Northern New England, we are interested in developing early maturing quinoa with short stature, non-branched or few branches with minimum branch angle, and higher grain yield. Therefore, it is our ultimate interest to develop a linkage map of *C. ficifolium* that can be helpful to identify markers and genes related to all of the traits of our interest. The P and QC accessions of *C. ficifolium* were evaluated for agronomically relevant traits, which provided valuable results indicative of substantial hereditary differences in the flowering time, plant height, number of branches, and branch angles between the two parental accessions. The results for internode length were not consistent between two experiments and therefore this trait needs further studies. Our results suggest that the P accession is closer to our ideotype, with its early flowering, reduced branching, and shorter plant height as compared with the QC accession. However, the P accession also has a higher branching angle, which is undesirable as explained below.

Plant height, branch angle, and internode length had continuous distributions (normal or non-normal) in the F2 generation population. Similar continuous distribution patterns of plant height and branch angle were found in F2 generation populations of other plant species [31, 32]. Plant height and branch angle are considered as important traits contributing to the architecture of plants. A study done in barley found a direct association of plant height with the degree of lodging resistance [33]. Lodging can be defined as a condition where plants bend slightly or excessively during a particular developmental stage [33]. The lodging of plants leads to direct contact with the surrounding plants and/or the soil, which can cause an easier spread of diseases. It can also cause mold and rot development in the seeds of a plant due to contact of seed heads with the ground during seed maturation. Besides this, it also limits the space necessary for intercultural operation and harvesting [4]. Susceptibility to lodging also depends on the branch angle for the plants having a branching architecture. A smaller branch angle allows higher plant density and also determines the plant’s ability to grow and increase light capture efficiency of the plant [34]. A small branch angle also helps the plant to keep its branches standing whereas a greater branch angle increases the chance of lodging or breaking of branches. Therefore, the development of an F2 population segregating for plant height and branch angle is a crucial accomplishment of this study.

Flowering time is one of the most important traits to be considered in a quinoa breeding program. Quinoa has a lengthy growing season, which is usually around 5 months [5]. This time period is favorable for growing quinoa in equatorial regions of South American countries as it allows seeds to mature and dry properly before harvest. But in places like New England region, frost starts as early as September and it reduces the seed quality, increases the drying cost, and makes the harvest more difficult due to moist seeds [4]. Shortening the flowering time (DAS) of quinoa could help eliminate these problems. We found bimodal distribution of flowering time in our *C. ficifolium* F2 population (Fig. 4). In contrast continuous distribution of flowering time was observed during a study done in buckwheat [31].

Our studies are in alignment to various breeding efforts in quinoa worldwide. Excessive branching is considered as a limitation for large scale agricultural production of quinoa [6]. Therefore breeding efforts of quinoa should focus on the development of short and non-branching quinoa that facilitate mechanical harvesting of plants [4]. During this study, we found a bimodal distribution pattern of the F2 generation for the number of branches. The bimodal distribution can be a result of a single gene influence on the trait, or it could also be due to the combined influence of a major QTL and some minor QTL. We didn’t find any literature focused towards genetic dissection of any of our traits of interest among any *Chenopodium* species, except for flowering time study in *C. ficifolium* [21]. A study was done in rapeseed (*Brassica napus*) to identify candidate genes related to branch angle with an objective to improve the plant architecture and yield of the crop [32], and it will be of future interest to look at the possible association of these genes with branch angle in *C. ficifolium* and quinoa.

### Trait correlations

We found a high positive correlation among flowering time, plant height, and the number of branches during our study in both the experiments, with one exception: Only moderate positive correlation was found between flowering time and plant height in the 2nd experiment. Based on our results, the plants flowering late were also the plants that tended to be taller and have a greater number of branches. Similar results were found in a study done in rapeseed (*Brassica napus*), in which plant height and plant branching were highly heritable and had a strong correlation [35]. Furthermore, in a study done among various quinoa cultivars, a significant correlation was found between seed yield per plant and other traits: days to flower bud formation, panicle length, plant height, biomass yield, days to pasty grain stage, days to milky grain, and days to physiological maturity [4].

### The *FLOWERING LOCUS T-LIKE* (*FTL*) marker locus and its amplicons

Our research took advantage of the prior development of the *FLOWERING LOCUS T-LIKE* (*FTL*) loci *FTL1* and *FTL2* as informative markers for confirmation of parentage in quinoa [20]. The published universal primers [26] were found to detect a very useful marker for both parentage and segregation analysis in our F2 generation population. The *FTL* primer pair generated multiple amplicons in the parental P and QC accessions, and each of the accessions had a unique amplicon (A1 or A2) that facilitated the identification of true hybrids, the predicted banding patterns of which differed from those of progeny from accidental selfing. Overall, our results were indicative that bands A1 and A2 represent alternate alleles of a single marker locus *FTL1* that is segregating normally in our study population. This outcome also provided a valuable foundation to utilize the *FTL1* marker locus to analyze marker-trait association, as is discussed in detail below.

Due to its potential usefulness, a better understanding of *FTL1* amplicon diversity was desired. Generation and comparison of amplicon sequences revealed that the P accession band A1 had some unique sequences totaling 1250 bp, possibly due to insertion, in the intron 3 region that were not found in the *FTL1* intron of our QC accession, as well as a previously studied *C. ficifolium* and a quinoa accession [20]. The QC accession also had a total of 227 bp of unique sequences in the same intron that was absent only in the P accession. Previous study by other authors [20] have also found insertion of 1500 bp in the accessions of quinoa and *C. berlandieri* which were absent in only one accession of *C. berlandieri*. The authors [20] found that the quinoa inserts contained long terminal repeat (LTR) sequences and therefore suspected the role of retrotransposons in molecular evolution of the *FTL1* introns. However, the intron 3 insertions found in the P accession and quinoa do not match. These results show that the *FTL1* intron 3 has been subject to dynamic evolution in these *Chenopodium* species due to frequent insertion and deletion as is also suggested by previous authors [20].

The P and QC accessions in general have a substantial difference in the introns as well as the promoter region of the *FTL1* gene. The QC accession had a deletion in a potential promoter site. The P accession had two unique non-synonymous substitutions in the CDS of exon 1 of *FTL1* gene. These non-synonymous substitutions could influence a differential expression in the *FTL1* gene of P accession causing the plants to flower earlier than the QC plants. This speculation can be further investigated by gene editing through mutation in these SNP sites. Also, we found that quinoa has three non-synonymous substitutions in the fourth exon of the *FTL1* gene that are not found in our P and QC accessions or in a previously studied accession of *C. ficifolium* [21].

### Association of *FTL1* genotype and trait variation

Upon genotyping of the studied F2 population, the *FTL1* amplicons A1 and A2 that were unique to the P and QC accessions, respectively, were found to be associated with three traits of interest: flowering time, plant height, and to a lesser extent number of branches. For instance, the A2 amplicon was only found in the F2 individuals that were late flowering, and the individuals possessing either A1 or both A1 and A2 were earlier flowering. It is noteworthy that the *FTL1* gene has been described as a floral activator/regulator in some *Chenopodium* studies [20, 26]. Thus, the possibility must be considered that the *FTL1* locus in *C. ficifolium* is more than just an associated marker: our results suggest that the alternate A1 and A2 *FTL1* alleles may themselves have differential functional influence on flowering time, plant height, and/or number of branches. Such a multifaceted role of a flowering gene has been reported in several studies [36] where the *FT-like* protein has been identified to regulate other developmental processes of plants such as fruit set, vegetative growth, stomatal opening control, and tuberization. A study done in Arabidopsis found *FLOWERING LOCUS T* (*FT*) gene to control lateral shoot outgrowth [37] . During that study, the authors also found that the Arabidopsis mutants for *FT* and *TWIN SISTER OF FT* (*TSF*, paralog of *FTL* gene) had a delay in the onset of outgrowth as well as a reduction of the growth rate. Therefore, the flowering gene indeed could be responsible for governing all of its correlated traits even in *C. ficifolium*. Alternatively, the *FTL1* gene might directly influence only one or two of these traits, while the correlation of the other trait(s) is a physiological or developmental byproduct of the primarily influenced trait[s]. It is also possible that the traits are influenced by an unknown neighbor gene and the *FTL1* gene is just a linked marker, albeit a very useful one.

## Conclusion

An F2 population segregating for flowering time, plant height, the number of branches, branch angle, and internode length was developed in *C. ficifolium*. Marker-trait association was identified between the *FLOWERING LOCUS T-LIKE 1* (*FTL1*) gene marker and three correlated phenotypic traits: flowering time, plant height, and the number of branches. The *FTL1* gene of *C. ficifolium* accessions P and QC had deletional differences in the promoter region and SNPs were also identified in the coding sequence of the gene. These genetic divergences among the accessions can be further investigated in their relation to the expression of the *FTL1* gene and their potential influence on the flowering behavior and architecture of plants. The germplasm we have developed will provide a useful system for studying the influence of the *FTL1* gene on these traits. As yet we do not know whether the *FTL1* gene will have similar influence in the tetraploid relative quinoa. However, this possibility warrants investigation. Further studies through linkage mapping on the developed F2 population could be helpful to identify other markers related to various traits of interest. We anticipate that our findings will be helpful for studies not only in *C. ficifolium* and *C. quinoa* but also to other important *Chenopodium* species.

## Supplementary Information

**Additional file 1**: **Figure S1.** *FTL* amplicons in parents and hybrids. Gel electrophoresis result of identified hybrid plants along with the parents and controls from P x QC crosses. *FTL* gene primers were used for the PCR amplification and a 1Kb^+^ DNA ladder was used for the amplicon size identification in the first lane. True hybrids have both diagnostic bands, A1 and A2 (arrows). This image has been cropped. The uncropped image is provided in Figure S5-Additional file 2. **Figure S2.** *FTL* amplicons segregating in F2 population. Gel electrophoresis of *FTL* amplicons from P and QC parental plants, three putative hybrids (Ph), and the 25 F2 plants in the 1^st^ experiment. The F2 plants are numbered from 1 to 25 in series from top and bottom gels and are arranged according to the flowering time (DAS). The positions of the diagnostic A1 and A2 bands are indicated by arrows to the left of the top gel. The lengths of the 1Kb+ DNA ladder bands used in both gels is represented in bp at the left of the bottom gel. This image has been cropped. The uncropped image is provided in Figure S6-Additional file 2. **Figure S3.** *FTL* amplicons segregating in F2 population. Gel electrophoresis results of three P, two QC, and 40 F2 individuals grown in the 2^nd^ Experiment. The F2 plants are numbered from 1 to 40 in series and are arranged according to the flowering time. The plants were genotyped using the *FTL* locus marker. 1kb + DNA ladder was used for the amplicon size identification in first lane. This image has been cropped. The uncropped image is provided in Figure S7-Additional file 2. **Figure S4.** Cloning and gel extraction of *FTL* amplicons. Gel electrophoresis of cloned amplicons of *FTL* marker system. B, A2, C, and D (left), and the gel extracted amplicon A1 (right) of the *FTL* marker system. The P and QC lanes show the parental accessions amplicons as controls. The lengths of the 1Kb+ DNA ladder bands used in both gels is represented in bp at the left. This image has been cropped. The uncropped image is provided in Figure S8-Additional file 2.

**Additional file 2**: **Figure S5.** *FTL* amplicons in parents and hybrids. Gel electrophoresis result of identified hybrid plants along with the parents and controls from P x QC crosses. *FTL* gene primers were used for the PCR amplification and a 1Kb^+^ DNA ladder was used for the amplicon size identification in the first lane. True hybrids have both diagnostic bands, A1 and A2 (arrows). This is an uncropped image. The cropped image is provided in Figure S1-Additional file 1. **Figure S6.** *FTL* amplicons segregating in F2 population. Gel electrophoresis of *FTL* amplicons from P and QC parental plants, three putative hybrids (Ph), and the 25 F2 plants in the 1^st^ experiment. The F2 plants are numbered from 1 to 25 in series from top and bottom gels and are arranged according to the flowering time (DAS). The positions of the diagnostic A1 and A2 bands are indicated by arrows to the left of the top gel. The lengths of the 1Kb+ DNA ladder bands used in both gels is represented in bp at the left of the bottom gel. This is an uncropped image. The cropped image is provided in Figure S2 - Additional file 1. **Figure S7.** *FTL* amplicons segregating in F2 population. Gel electrophoresis results of three P, two QC, and 40 F2 individuals grown in the 2^nd^ Experiment. The F2 plants are numbered from 1 to 40 in series and are arranged according to the flowering time. The plants were genotyped using the *FTL* locus marker. 1kb + DNA ladder was used for the amplicon size identification in first lane. This is an uncropped image. The cropped image is provided in Figure S3-Additional file 1. **Figure S8.** Cloning and gel extraction of *FTL* amplicons. Gel electrophoresis of cloned amplicons of *FTL* marker system. B, A2, C, and D (top gel), and the gel extracted amplicon A1 (bottom gel) of the *FTL* marker system. The P and QC lanes show the parental accessions amplicons as controls. The Lane 2 (bottom gel) represents A1 amplicon isolation results from a separate gel. The lane A1 template was used for sequencing of the A1 amplicon. The lanes X and Y (top gel) represent results from a different *FTL* marker study. The lengths of the 1Kb+ DNA ladder bands used in both gels is represented in bp at the left of both gels. This is an uncropped image. The cropped image is provided in Figure S4-Additional file 1.

**Additional file 3**: **Table S1**-C: Evaluation of flowering time difference between Portsmouth and Quebec accessions. **Table S2.** Goodness of fit tests. Chi-Square Goodness of fit tests for segregation of *FTL1* genotypes, A1A1, A1A2, and A2A2 among the F2 populations of 1^st^ and 2^nd^ experiment. The table also shows the results for the Chi-Square homogeneity tests on the F2 populations of the two experiments. **Table S3**. BLASTn result of all FTL amplicons to the Cq_PI614886_V1 chromosome assembly of quinoa. **Table S4.** T-tests. T-Test (Independent sample with unequal variance) results for comparison between P and QC accessions for flowering time, plant height, number of branches, branch angle, and internode length in the two experiments. The mean (de-transformed), df, t-ratio, and the *p*-value of each test is included. **Table S5**. Multivariate correlation analysis of traits phenotyped in 1st and 2nd experiments. **Table S6**. Pairwise t-tests on *FTL1* genotypes of 1st and 2nd experiments. **Table S7**. Unique SNPs identified in *C. ficifolium* accessions, Portsmouth and Quebec, in reference to *FTL1* gene in B-subgenome of quinoa.

## Data Availability

Phenotypic and variant calling data are available in the Supplementary files. The amplicons contig are available publicly in the GenBank under accession number MW251945 to MW251949. The sequencing reads are available in the Sequence Read Archive (SRA) database (GenBank: PRJNA678370). A preliminary report on this work has been presented in the Master’s thesis of M. Subedi [24].

## Abbreviations

*SOS1*: *SALT OVERLY SENSITIVE 1*
*FTL*: *FLOWERING LOCUS T- LIKE*
SNP: Single nucleotide polymorphism
CTAB: Cetyltrimethylammonium bromide
PCR: Polymerase chain reaction
CRD: Completely randomized design
DAS: Days after sowing
CDS: Coding sequence
