# Supplementary material for: Developing Chenopodium ficifolium as a potential B genome diploid model system for genetic characterization and improvement of allotetraploid quinoa (Chenopodium quinoa)
Source: BMC Plant Biol. 2021 Oct 25;21:490. doi: 10.1186/s12870-021-03270-5 (PMC8543794; doi:10.1186/s12870-021-03270-5)
Supplement: Supplementary file 2 — Additional file 2: Figure S5. FTL amplicons in parents and hybrids. Gel electrophoresis result of identified hybrid plants along with the parents and controls from P x QC crosses. FTL gene primers were used for the PCR amplification and a 1Kb+ DNA ladder was used for the amplicon size identification in the first lane. True hybrids have both diagnostic bands, A1 and A2 (arrows). This is an uncropped image. The cropped image is provided in Figure S1-Additional file 1. Figure S6. FTL amplicons segregating in F2 population. Gel electrophoresis of FTL amplicons from P and QC parental plants, three putative hybrids (Ph), and the 25 F2 plants in the 1st experiment. The F2 plants are numbered from 1 to 25 in series from top and bottom gels and are arranged according to the flowering time (DAS). The positions of the diagnostic A1 and A2 bands are indicated by arrows to the left of the top gel. The lengths of the 1Kb+ DNA ladder bands used in both gels is represented in bp at the left of the bottom gel. This is an uncropped image. The cropped image is provided in Figure S2 - Additional file 1. Figure S7. FTL amplicons segregating in F2 population. Gel electrophoresis results of three P, two QC, and 40 F2 individuals grown in the 2nd Experiment. The F2 plants are numbered from 1 to 40 in series and are arranged according to the flowering time. The plants were genotyped using the FTL locus marker. 1kb + DNA ladder was used for the amplicon size identification in first lane. This is an uncropped image. The cropped image is provided in Figure S3-Additional file 1. Figure S8. Cloning and gel extraction of FTL amplicons. Gel electrophoresis of cloned amplicons of FTL marker system. B, A2, C, and D (top gel), and the gel extracted amplicon A1 (bottom gel) of the FTL marker system. The P and QC lanes show the parental accessions amplicons as controls. The Lane 2 (bottom gel) represents A1 amplicon isolation results from a separate gel. The lane A1 template was used for sequencing of [file 12870_2021_3270_MOESM2_ESM.docx]

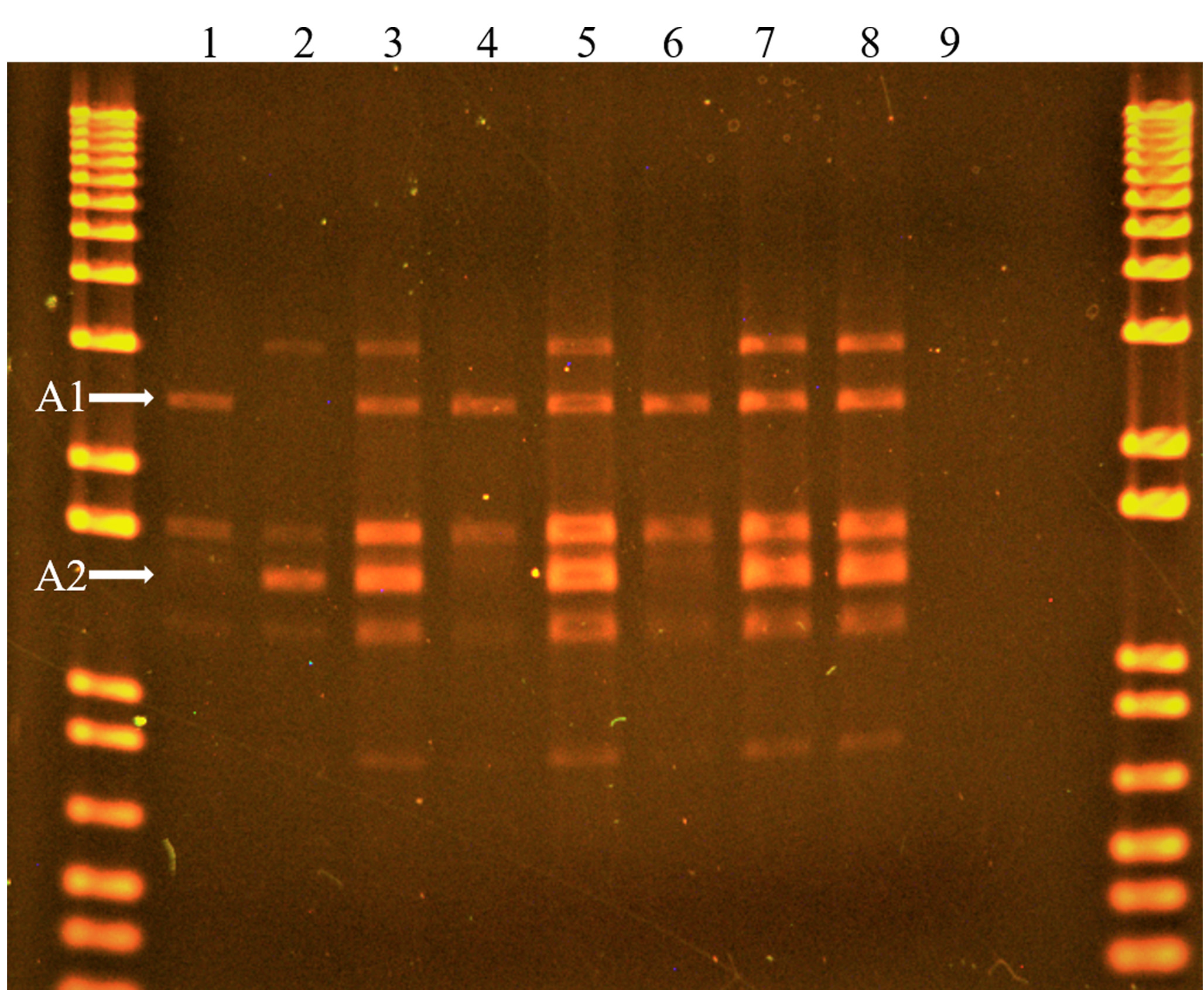


**Figure S5-Additional file 2: *FTL* amplicons in parents and hybrids**. Gel electrophoresis result of identified hybrid plants along with the parents and controls from P x QC crosses. *FTL* gene primers were used for the PCR amplification and a 1Kb^+^ DNA ladder was used for the amplicon size identification in the first lane. True hybrids have both diagnostic bands, A1 and A2 (arrows). This is an uncropped image. The cropped image is provided in Figure S1-Additional file 1.

Lane labels:

Left and right: 1Kb^+^ DNA ladder
Lane 1: Portsmouth (P.1) (maternal parent)
Lane 2: Quebec (QC4.1) (paternal parent)
Lane 3: Putative hybrid A “P1F1A” -True hybrid
Lane 4: Putative hybrid B “P1F1B”
Lane 5: Putative hybrid C “P1F1C” -True hybrid Lane 6: Putative hybrid D “P1F1D”
Lane 7: Mixed template
Lane 8: Mixed PCR product
Lane 9: Control (no template added)


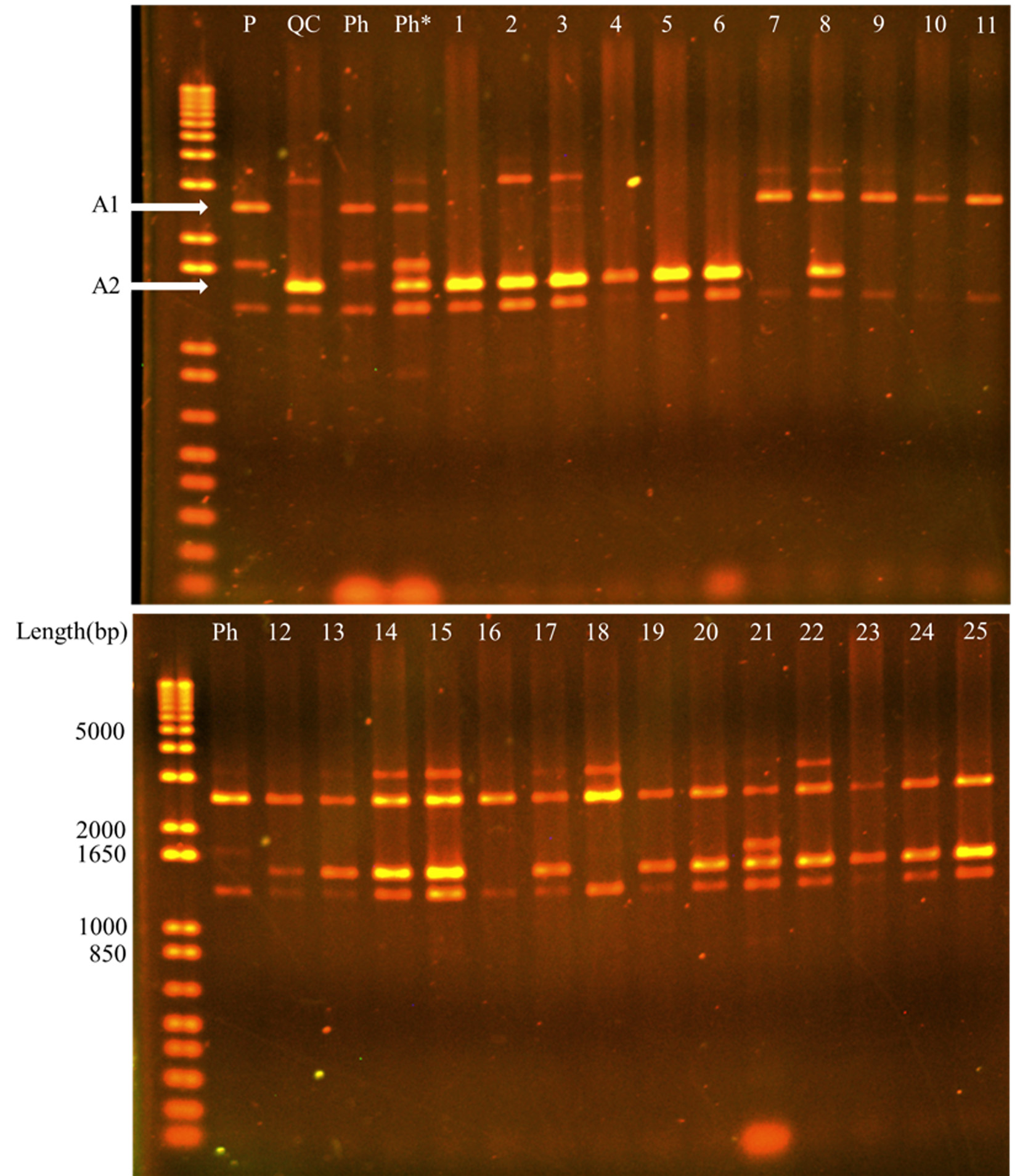


**Figure S6-Additional file 2: *FTL* amplicons segregating in F2 population.**

Gel electrophoresis of *FTL* amplicons from P and QC parental plants, three putative hybrids (Ph), and the 25 F2 plants in the 1^st^ experiment. The F2 plants are numbered from 1 to 25 in series from top and bottom gels and are arranged according to the flowering time (DAS). The positions of the diagnostic A1 and A2 bands are indicated by arrows to the left of the top gel. The lengths of the 1Kb+ DNA ladder bands used in both gels is represented in bp at the left of the bottom gel. This is an uncropped image. The cropped image is provided in Figure S2 - Additional file 1.

Lane labels:

Left: 1 kb+ DNA ladder

P and QC: Portsmouth and Quebec plants, respectively
Ph: Putative hybrids

1 to 6: F2 plants flowering at greater than 28 DAS
7 to 11: F2 plants flowering at 21 DAS

12 to 20: F2 plants flowering at 23 DAS

21 to 25: F2 plants flowering at 25 to 29 DAS


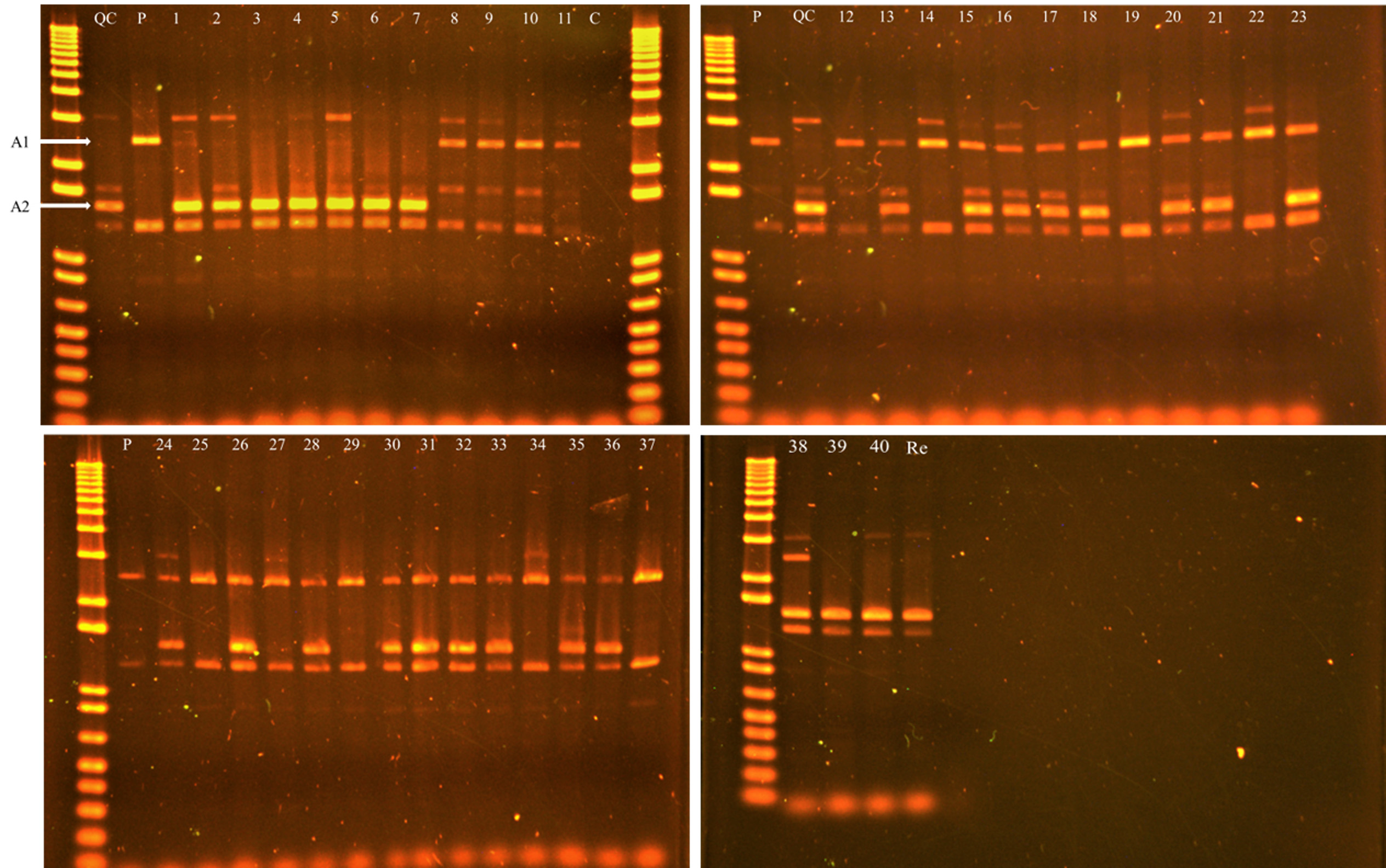


**Figure S7-Additional file 2: *FTL* amplicons segregating in F2 population.** Gel electrophoresis results of three P, two QC, and 40 F2 individuals grown in the 2^nd^ Experiment. The F2 plants are numbered from 1 to 40 in series and are arranged according to the flowering time. The plants were genotyped using the *FTL* locus marker. 1kb + DNA ladder was used for the amplicon size identification in first lane. This is an uncropped image. The cropped image is provided in Figure S3-Additional file 1.

Lane labels:

Left: 1 kb+ DNA ladder. Also present in the right of first gel (top left).
P and QC: Portsmouth and Quebec plants, respectively
1 to 7: F2 plants flowering at greater than 28 DAS
8 to 16: F2 plants flowering at 16-18 DAS
17 to 38: F2 plants flowering at 20-23 DAS

39 & 40: F2 plants flowering at 27 DAS

Re: Same PCR product as in lane 40


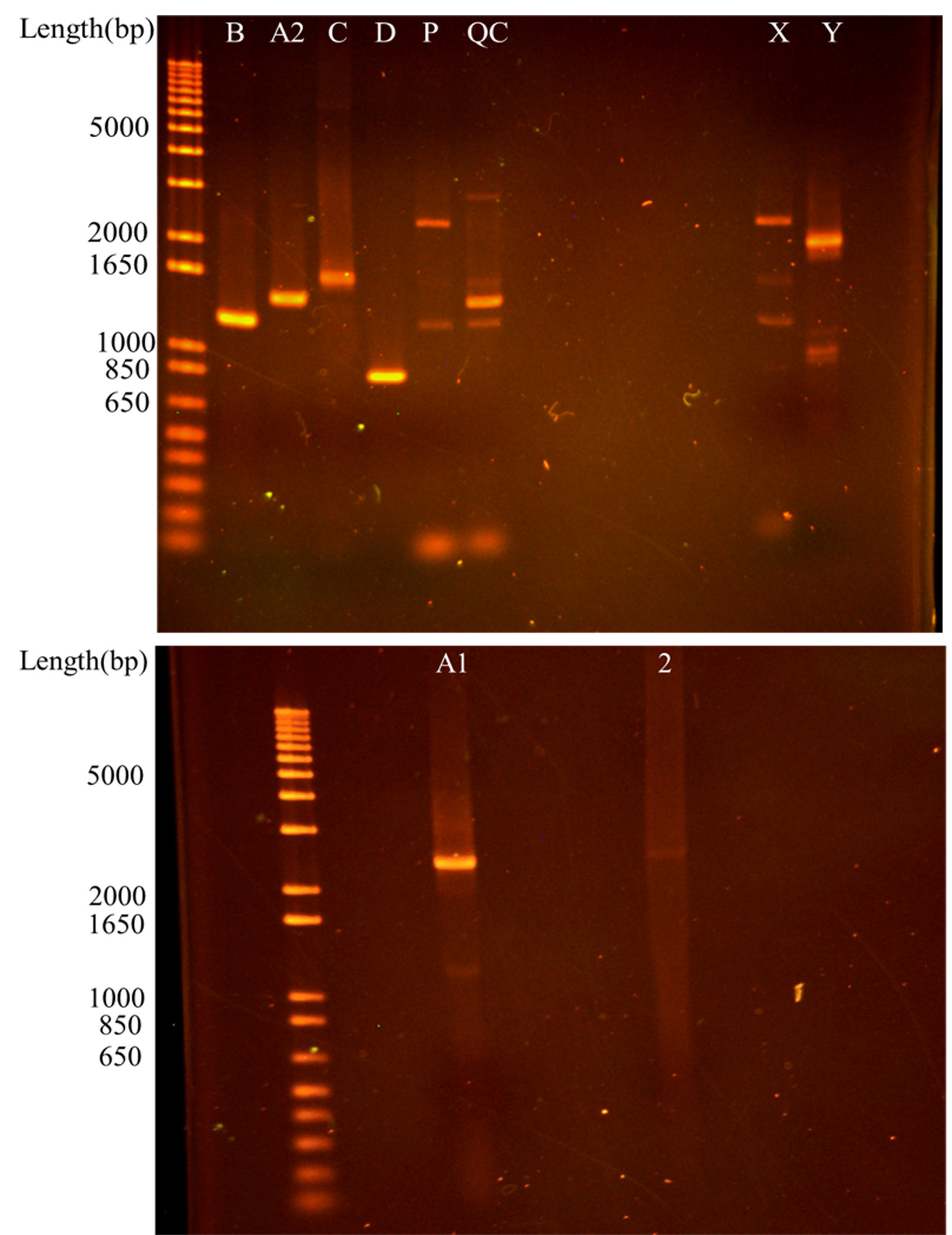


**Figure S8-Additional file 2: Cloning and gel extraction of *FTL* amplicons.** Gel electrophoresis of cloned amplicons of *FTL* marker system. B, A2, C, and D (top gel), and the gel extracted amplicon A1 (bottom gel) of the *FTL* marker system. The P and QC lanes show the parental accessions amplicons as controls. The Lane 2 (bottom gel) represents A1 amplicon isolation results from a separate gel. The lane A1 template was used for sequencing of the A1 amplicon. The lanes X and Y (top gel) represent results from a different *FTL* marker study. The lengths of the 1Kb+ DNA ladder bands used in both gels is represented in bp at the left of both gels. This is an uncropped image. The cropped image is provided in Figure S4-Additional file 1.
