# Supplementary material for: Developing Chenopodium ficifolium as a potential B genome diploid model system for genetic characterization and improvement of allotetraploid quinoa (Chenopodium quinoa)
Source: BMC Plant Biol. 2021 Oct 25;21:490. doi: 10.1186/s12870-021-03270-5 (PMC8543794; doi:10.1186/s12870-021-03270-5)
Supplement: Supplementary file 3 — Additional file 3: Table S1-C: Evaluation of flowering time difference between Portsmouth and Quebec accessions. Table S2. Goodness of fit tests. Chi-Square Goodness of fit tests for segregation of FTL1 genotypes, A1A1, A1A2, and A2A2 among the F2 populations of 1st and 2nd experiment. The table also shows the results for the Chi-Square homogeneity tests on the F2 populations of the two experiments. Table S3. BLASTn result of all FTL amplicons to the Cq_PI614886_V1 chromosome assembly of quinoa. Table S4. T-tests. T-Test (Independent sample with unequal variance) results for comparison between P and QC accessions for flowering time, plant height, number of branches, branch angle, and internode length in the two experiments. The mean (de-transformed), df, t-ratio, and the p-value of each test is included. Table S5. Multivariate correlation analysis of traits phenotyped in 1st and 2nd experiments. Table S6. Pairwise t-tests on FTL1 genotypes of 1st and 2nd experiments. Table S7. Unique SNPs identified in C. ficifolium accessions, Portsmouth and Quebec, in reference to FTL1 gene in B-subgenome of quinoa. [file 12870_2021_3270_MOESM3_ESM.zip › Additional file 3_Table S4_Parental trait diversity.docx]

**Table S4-Additional file 3: T-tests.** T-Test (Independent sample with unequal variance) results for comparison between P and QC accessions for flowering time, plant height, number of branches, branch angle, and internode length in the two experiments. The mean (de-transformed), df, t-ratio, and the p-value of each test is included.

| **Trait** | **Experiment** | **Accession** | **Mean** | **df** | **t-ratio** | **p-value** |
| --- | --- | --- | --- | --- | --- | --- |
| Flowering time (DAS) | 1 | P | 23.8 | 8 | 8.69 | <0.0001 |
|  |  | QC | 34.7 |  |  |  |
|  | 2 | P | 23.4 | 4 | 6.47 | 0.0025 |
|  |  | QC | 30.3 |  |  |  |
| Plant height (cm) | 1 | P | 41.4 | 10 | 12.7 | <0.0001 |
|  |  | QC | 68.2 |  |  |  |
|  | 2 | P | 54.3 | 6 | 5.4 | 0.0016 |
|  |  | QC | 68.7 |  |  |  |
| Number of branches | 1 | P | 15.0 | 12 | 1 5.9 | <0.0001 |
|  |  | QC | 25.5 |  |  |  |
|  | 2 | P | 17.7 | 5 | 9.98 | 0.0001 |
|  |  | QC | 27.7 |  |  |  |
| Branch angle (degree) | 1 | P | 78.0 | 9 | -7.68 | <0.0001 |
|  |  | QC | 61.7 |  |  |  |
|  | 2 | P | 79.4 | 4 | -7.68 | 0.0019 |
|  |  | QC | 61.5 |  |  |  |
| Internode length (cm) | 1 | P | 2.8 | 11 | -1.03 | 0.32 |
|  |  | QC | 2.6 |  |  |  |
|  | 2 | P | 3.1 | 4 | -3.7 | 0.016 |
|  |  | QC | 2.5 |  |  |  |
